# An invasive *Haemophilus influenzae* serotype b infection in an Anglo-Saxon plague victim

**DOI:** 10.1186/s13059-021-02580-z

**Published:** 2022-02-03

**Authors:** Meriam Guellil, Marcel Keller, Jenna M. Dittmar, Sarah A. Inskip, Craig Cessford, Anu Solnik, Toomas Kivisild, Mait Metspalu, John E. Robb, Christiana L. Scheib

**Affiliations:** 1 Estonian Biocentre, Institute of Genomics, University of Tartu, Riia 23B, 51010 Tartu, Estonia; 2 McDonald Institute for Archaeological Research, University of Cambridge, Downing Street, Cambridge, CB2 3ER UK; 3 Department of Archaeology, University of Aberdeen, St. Mary’s, Elphinstone Road, Aberdeen, Scotland AB24 3UF UK; 4 School of Archaeology and Ancient History, University of Leicester, University Road, Leicester, LE1 7RH UK; 5 Cambridge Archaeological Unit, University of Cambridge, 34 A&B Storey’s Way, Cambridge, CB3 0DT UK; 6 Core Facility, Institute of Genomics, University of Tartu, Riia 23B, 51010 Tartu, Estonia; 7 Department of Human Genetics, KU Leuven, Herestraat 49, B-3000 Leuven, Belgium; 8 Department of Archaeology, University of Cambridge, Downing Street, Cambridge, CB2 3DZ UK; 9 St John’s College, University of Cambridge, St John’s Street, Cambridge, CB2 1TP UK

**Keywords:** *Haemophilus influenzae*, *Yersinia pestis*, Plague, aDNA, Ancient DNA, Microbial genomics, Osteology, Serotype b, Pathogen genomics, Paleogenomics

## Abstract

**Background:**

The human pathogen *Haemophilus influenzae* was the main cause of bacterial meningitis in children and a major cause of worldwide infant mortality before the introduction of a vaccine in the 1980s. Although the occurrence of serotype b (Hib), the most virulent type of *H. influenzae*, has since decreased, reports of infections with other serotypes and non-typeable strains are on the rise. While non-typeable strains have been studied in-depth, very little is known of the pathogen’s evolutionary history, and no genomes dating prior to 1940 were available.

**Results:**

We describe a Hib genome isolated from a 6-year-old Anglo-Saxon plague victim, from approximately 540 to 550 CE, Edix Hill, England, showing signs of invasive infection on its skeleton. We find that the genome clusters in phylogenetic division II with Hib strain NCTC8468, which also caused invasive disease. While the virulence profile of our genome was distinct, its genomic similarity to NCTC8468 points to mostly clonal evolution of the clade since the 6th century. We also reconstruct a partial *Yersinia pestis* genome, which is likely identical to a published first plague pandemic genome of Edix Hill.

**Conclusions:**

Our study presents the earliest genomic evidence for *H. influenzae*, points to the potential presence of larger genomic diversity in the phylogenetic division II serotype b clade in the past, and allows the first insights into the evolutionary history of this major human pathogen. The identification of both plague and Hib opens questions on the effect of plague in immunocompromised individuals already affected by infectious diseases.

**Supplementary Information:**

The online version contains supplementary material available at 10.1186/s13059-021-02580-z.

## Background

*Haemophilus influenzae* is an opportunistic bacterial pathogen that colonizes the human nasopharynx. The gram-negative bacterium is restricted to human hosts and can be divided into two main groups characterized by the expression of antigenically distinct polysaccharide capsules: encapsulated types (serotypes a–f) and non-encapsulated types, also called non-typeable strains (NTHi). The pathogen causes a wide spectrum of disease, both acute and chronic, the severity and clinical phenotype of which can depend on the type and genetic makeup of the *H. influenzae* strain colonizing the organism [1]. Due to the high rates of asymptomatic carriage, *H. influenzae* is considered part of the commensal microbiome of the upper respiratory tract when detected in the nasopharynx of healthy individuals [2, 3], from where it can spread to cause disease via respiratory droplets. Common illnesses stemming from an *H. influenzae* infection are pneumonia, otitis media, conjunctivitis, and sinusitis.

*H. influenzae* can also cause acute/invasive diseases such as septicemia, meningitis, epiglottitis, cellulitis, osteomyelitis, and septic arthritis, which necessitate infection of the bloodstream and hematogenous dissemination [2, 4]. The bulk of invasive cases occur in children under the age of five, but it has been known to occur in all age groups, particularly in immunocompromised individuals [2]. These severe cases have generally been attributed to the most pathogenic type of *H. influenzae*, serotype b (Hib), which is estimated to have been responsible for more than 95% of all invasive cases and was the main cause for bacterial meningitis in children prior to the introduction of a conjugate vaccine in the late 1980s [1, 5]. Today, NTHi strains and other serotypes have filled the gap left by the near eradication of Hib in vaccinated populations and are causing increasing numbers of infections [1, 6].

The pathogen is speculated to have been a major player in infantile health worldwide before its first identification in 1892 from a throat swab. It was then believed to be the causative pathogen for the flu, thus its species epitheton “*influenzae*”, an error that was only corrected in 1933, when the influenza virus was identified [2, 7, 8]. Prior to these events, the wide breadth of symptoms and degrees of severity of *H. influenzae* infections make the identification of early records of the disease in historical sources nearly impossible. Additionally, commensal carriage and non-invasive infections would be unlikely to leave traces in the osteological record, and while invasive infections might leave indistinct pathological lesions, they would be almost impossible to diagnose on paleopathological features alone.

*Yersinia pestis*, the causative agent of plague, is a gram-negative bacterium that evolved only about 30,000–50,000 years ago [9] from its ancestor *Yersinia pseudotuberculosis* through a distinctive pattern of acquisition and loss of specific virulence factors [10]. In general, *Y. pestis* causes acute and severe infections in humans, commonly associated with septicemia. Its lethality is estimated to be ca. 50% for bubonic plague and close to 100% for pneumonic plague without antibiotic treatment [11]. Traditionally, the history of plague is divided into the first pandemic starting with the Justinianic plague in 541 CE in the Eastern Mediterranean with recurrent outbreaks until 750 CE, the second pandemic from the fourteenth to the eighteenth centuries CE including the Black Death in 1346–1353 CE, and the third pandemic, which started in the mid-nineteenth century and is responsible for sporadic cases and local epidemics in Asia, Africa, and North and South America until today [12].

In this study, we present a case of co-infection of *H. influenzae* serotype b and *Yersinia pestis* identified via ancient DNA (aDNA) analysis. The bacteria were detected in a tooth sample from a non-adult skeleton from the Anglo-Saxon cemetery of Edix Hill (Barrington A), associated with the first plague pandemic (541–750 CE). This is the second case of plague co-infection in archeological samples reported recently [13]. The site of Edix Hill, which has yielded multiple plague genomes [14], illustrates how plague affected whole populations already afflicted by other diseases and might have been the final blow for already immunocompromised individuals. Additionally, this is the earliest evidence for Hib in human populations. It is likely also the first detected instance of *H. influenzae* in aDNA datasets as sequences previously described from dental calculus more closely matched *Haemophilus parainfluenzae*, a pathogen considered commensal in the oropharynx (see Additional file 1).

Not much is known of the early evolutionary history of the species *H. influenzae*, and today, both typeable and non-typeable strains are split into non-geographically and non-epidemiologically definable clades. Our study points to the potential presence of larger genomic diversity in the phylogenetic division II serotype b clade, which is ostensibly no longer represented in recent diversity. Strain HI-EDI064 shows clear genomic differences to its genomically closest strain and has a distinct virulence profile, which hints at the presence of fimbriae on the bacterium’s cell walls. Our multidisciplinary data further enabled us to reconstruct the pathology of the non-adult plague victim based on osteological and genomic data. Finally, it allowed us to consider the potential implications of a co-infection with *Y. pestis* both to the competing pathogen and the host.

## Results

### Archeology and sample

The site of Edix Hill (Barrington, Cambridgeshire, UK) is an Anglo-Saxon cemetery dating between 500 and 650 CE [15]. We sampled teeth from two individuals recovered from an unphased double burial (grave 85) (Fig. S1a). The grave housed an adult male skeleton (25–35 years) (Sk 447A, EDI086/PSN625) with antemortem cranial trauma and a non-adult skeleton (6 years) (Sk 447B, EDI064/PSN604) showing non-pathognomonic signs of disease described below. They were excavated from a shallow grave, which seems to have been disturbed on multiple occasions leading to the recovery of partial skeletons (Fig. S1b). The deposition history of the grave could not be reconstructed based on site stratigraphy [15]. However, the recovery of *Y. pestis* reads from both skeletons is suggestive of their contemporaneity. Radiocarbon dating of individual EDI064 (416-541 calAD) further supports an association with the first wave of the Justinianic plague 541–544 CE (see Additional file 1).

### Osteology

The skeleton of EDI064, first recorded by Duhig [16], was approximately 50–75% complete (Fig. S1b) with the preservation of surviving skeletal elements ranging from fair to good. The genetic analysis identifies the individual’s sex as male (see below for details). The majority of the epiphyses were not present, although those making up the knee survived. While the majority of the lower limbs were absent, 90% of the left femur and the proximal end of the tibia were present. Over 80% of the cranial vault was present, but most of the facial bones were missing. Taphonomic damage to some of the postcranial elements complicated differential diagnosis as the full extent of the pathologies was impossible to discern.

No evidence of subperiosteal new bone formation was observed on the upper limbs or the axial skeleton. Subperiosteal new bone formation and porosity were present on the body of the left calcaneus (Fig. S1d). The incompleteness of the human skeletal remains limits the identification of subperiosteal new bone formation on the long bones of the lower limbs, although none was present on the left femur. There was no evidence for respiratory infection in the form of visceral rib lesions or lytic destruction of the vertebral bodies. There was no porotic hyperostosis, spondylolysis, vitamin D or C deficiency, or dental enamel hypoplasia. Cribra orbitalia and sinusitis were unobservable.

Both patellae were ankylosed to the anterior surface of the distal femoral epiphysis (Fig. S1c). This was confirmed with plain X-rays of the right distal femoral epiphysis which show an area of increased radiopacity indicating ankylosis (Fig. S2). On both distal femoral epiphyses, osteochondral defects were present on the medial and lateral condyles, mostly affecting the inferior and posterior surfaces (Fig. S1c and e). No evidence of these defects were seen on the proximal epiphyses of the right tibia (the left was unobservable). These lesions indicate localized areas of damage to the cartilage and underlying bone. The appearance of these cortical defects differs from those that occur in juvenile osteochondritis dissecans (OD), which is caused by the detachment of cartilage from the bone surface. OD lesions are usually discrete and have porous well-defined margins unlike what is observed in this individual where multiple lesions are present. These lesions are also inconsistent with juvenile idiopathic arthritis and juvenile-onset rheumatoid arthritis. Given that all the condyles are affected, and there is no other evidence for fracture, it is unlikely that this is related to a singular traumatic event. However, it is possible that the fusion of the patella affected his ability to flex his knee creating a reduced range of motion in the legs. This may have resulted in repetitive microtrauma while walking which may have caused the osteochondral defects observed. It is also possible that the soft tissue was damaged due to inflammation from invasive microorganisms which could have resulted in the cartilage being more prone to damage.

Bilateral patellofemoral ankylosis is very uncommon; there are very few published clinical cases, and to the best of our knowledge, no other archeological cases have ever been reported. The diagnosis of the skeletal lesions was undertaken with reference to Buikstra 2019 [17], Lewis 2017 [18], Rodgers and Waldron 1995 [19], and Waldron 2009 [20] as well as with reference to the clinical literature. The diagnosis of the observed skeletal lesions was complicated by a number of factors, including some that are not found in clinical cases. Specifically, the differential diagnosis of the observed lesions was made more difficult by the incompleteness of the skeleton and the lack of soft tissues.

Given the age of this individual, the observed lesions are unlikely to have been caused by seronegative spondyloarthropathies such as ankylosing spondylitis, psoriatic arthritis, reactive arthritis, and rheumatoid arthritis. As there is no evidence of skeletal trauma, osteomyelitis, or osteoarthritis, the ankylosis of the patella to the distal femoral epiphysis is likely to be the result of septic arthritis, a destructive arthropathy caused by infection, as bone fusion can occur [21].

### Metagenomics

Shotgun sequencing data from both individuals were analyzed metagenomically with Kraken2 [22] and KrakenUniq [23] using custom databases (see the “Methods” section). Both algorithms indicated the presence of *H. influenzae* and *Y. pestis* for individual EDI064 (KrakenUniq *E*-values of 0.015 and 0.006, respectively; see the “Methods” section). The presence of *Y. pestis* for the adult individual was tentatively confirmed by KrakenUniq (*E*-value = 0.005), but the assessment was based on only 75 *Y. pestis* reads and the coverage was low.

### Species validation

**Fig. 1:**
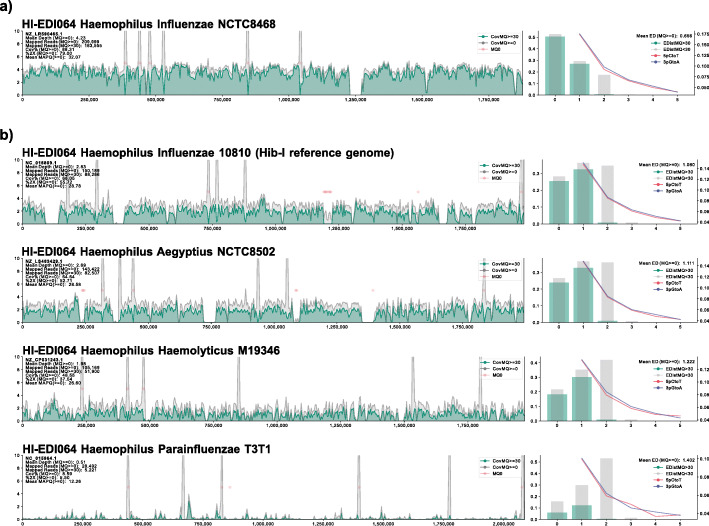
Non-competitive comparative mappings for the final reference sequence, the main serotype b reference and closely related *Haemophilus* species (from the phylogenetically closest to most distance to *H. influenzae*). Plots on the left show the depth of coverage across the genome, and plots on the right depict the deamination signatures (right axis; calculated using mapDamage2) and a histogram of the read edit distances (left axis)

**Fig. 2:**
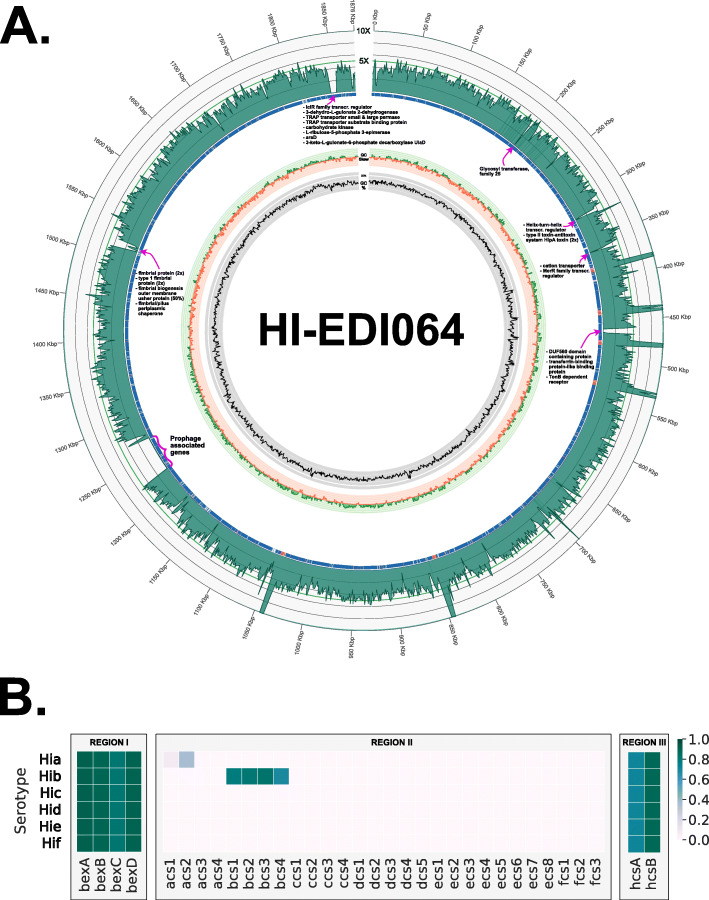
**A** Circos coverage plot of our mapping to NCTC8468. From the outer to the inner ring: depth of coverage, mappability, GC content, and GC Skew. Denoted with pink arrows are clear deletions in the alignment. **B** Results of the serotyping analysis for HI-EDI064 depicting coverage fractions for each interval of the serotyping databases. Coverage values are the maximum values observed among alleles for each gene

The identification of *H. influenzae* was validated by the presence of species-specific genes *fucAKLPU*, *pstACS*, *phoBR*, *pdxS*, and *dpxT* (Additional file 2: Table S3b) [24] as well as the presence of a *cap*-locus (Additional file 2: Table S4). Non-competitive mappings to reference genomes for *H. parainfluenzae*, *H. haemolyticus*, and *H. aegyptius* further confirmed our results at the genus level (see Fig. 1). Serotyping determined that the strain HI-EDI064 carried cap-locus region II genes consistent with serotype b (see Fig. 2b and Additional file 2: Table S4). Mappings to the reference sequences for *H. influenzae* serotypes a–f (Fig. S3) showed that the reference sequence for serotype f (KR949) had the lowest mean edit distance and highest coverage. Additionally, the presence of the gene *sodC* placed the ancient strain in phylogenetic division II [25, 26]. Our mapping to the sequence of strain NCTC8468 (NZ_LR590465.1) (see Fig. 2a and Fig. S3), a division II Hib carrying *sodC*, showed by far the best match, the highest depth of coverage, and the least number of missing intervals across the genome. Based on the depth of coverage of the NCTC8468 mapping, the sequence was used as a reference sequence throughout our analysis for *H. influenzae*.

The HI-EDI064 alignment to NCTC8468, a phylogenetic division II Hib isolated from the spinal fluids of a child suffering from meningitis in 1940 [27], has a mean depth of coverage of 4.23×, a mean fragment length of 37.8 bp, and a mean edit distance of 0.66, with 79% of the genome covered at least twice (Additional file 2: Table S5). Deamination was present with ~ 14% of reads estimated to be affected by C>T and G>A changes. Compared to NCTC8468, HI-EDI064 is missing a prophage-associated interval, which is unsurprising considering that studies have demonstrated that many *H. influenzae* genomes carry discrete intact phages, which make up a large portion of the species’ accessory genome [1] (see Fig. 2a). It also lacks coverage in a range of pili-associated intervals (1,512,864–1,519,532) while showing some coverage in haemagglutinating pili gene intervals.

### Phylogenetic reconstruction

**Fig. 3:**
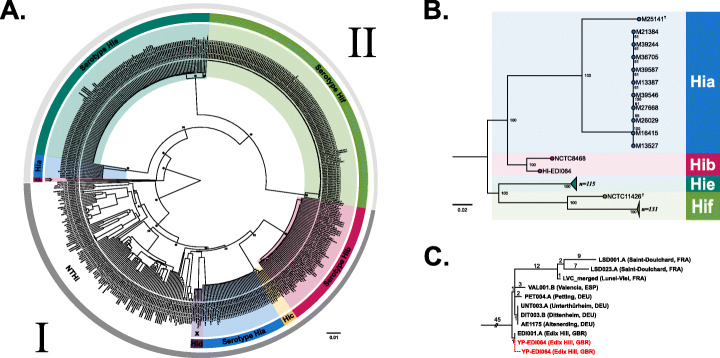
Phylogenies for *H. influenzae* and *Y. pestis*. **A** Maximum likelihood phylogeny of 492 modern and one ancient *H. influenza*e genomes. The tree is midpoint-rooted, and node support is based on 1000 bootstrap replicates in IQTREE-2. The main nodes with bootstrap values of 100 are marked with stars. The outer rings and roman numerals outline the two phylogenetic divisions. An arrow shows the position of our ancient genome, and the “x” denotes the presence of non-typable strains in serotype-specific clades. **B** Midpoint-rooted maximum likelihood phylogeny of 258 modern and one ancient *H. influenzae* phylogenetic division II genomes. Clades for serotypes e and f are collapsed. Genomes labeled with a cross are known recombinants [3]. Node support is based on 1000 bootstrap replicates in IQTREE-2. **C** Schematic tree of genomes of the first plague pandemic based on Keller et al. [14], showing two possible phylogenetic positions for YP-EDI064 (either identical with EDI001 or being directly derived from EDI001)

To determine the phylogenetic position of HI-EDI064, we used 493 *H. influenzae* genomes (Additional file 2: Table S1) and aligned them to the NCTC8468 assembly as our ancient genome showed a significant increase in coverage when using this reference genome. The species *H. influenzae* is characterized by significant genomic diversity, with the accessory genome making up more than half of its open pan-genome, and is known to acquire and lose genes during the infection process while maintaining its relative genome size [1]. Additionally, while the capsular clades are known to be largely clonal, the non-encapsulated strains have shown to be much more plastic, leading to large-scale recombination across all branches [1, 6]. We excluded the recombinant regions as well as the conserved/low complexity intervals from the analysis. We constructed a maximum likelihood phylogeny using the GTR+F+ASC+R3 model and 1000 standard bootstrap replicates as implemented in IQ-TREE2 [28]. Bootstrap values for almost all main nodes are excellent, and the serotype-specific clades, as well as the basal phylogenetic split between phylogenetic divisions I and II [1, 3, 29], are clearly differentiated (see Fig. 3a). As expected from previous analyses, HI-EDI064 clusters in phylogenetic division II basal to the clonal serotype a (Hia), e (Hie), and f (Hif) clades. The phylogenetically closest genomes are the reference genome NCTC8468, which is the only other phylogenetic division II Hib (Hib-II) genome on the tree, and the division II Hia clade. This clustering is further supported in our virulence analysis (see below). We also generated a maximum likelihood phylogeny of all 259 phylogenetic division II genomes, which allowed us to make use of more data. In this tree, we can observe clear serotype-specific clades with both Hib-II genomes clustering together (see Fig. 3b). Our SnpEff analysis yielded six SNPs with predicted high functional impact (Fig. S5 and Additional file 2: Table S6).

### Virulence analysis

**Fig. 4:**
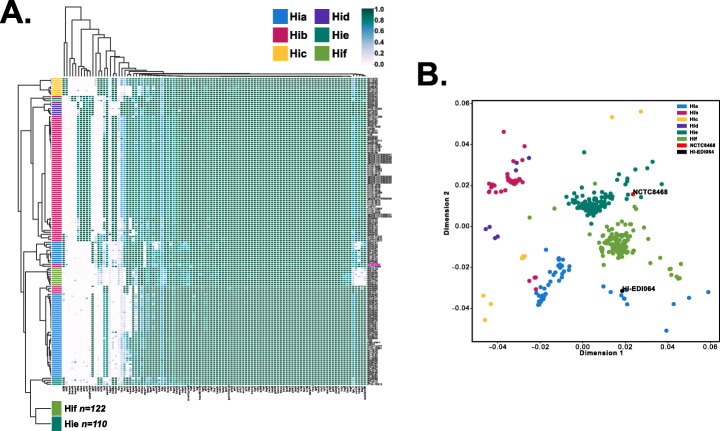
Virulence profile analysis for typable genomes. **A** Clustermap of coverage in virulence-associated gene intervals for typable genomes. Hie and Hif clusters are collapsed (see Fig. S4 for the full heatmap). The clustermap was generated using seaborn [108]. B Multidimensional scaling plot of capsular genomes based on the coverage in virulence-associated gene intervals

We computed a clustermap of coverage in virulence-associated gene intervals for capsular genomes and found that Hib-II genomes cluster closest to phylogenetic division II Hia (Hia-II) genomes (see Fig. 4). A notable difference is the absence of the liposaccharide-associated interval lgtA in Hib-II genomes. Compared to phylogenetic division I Hib (Hib-I), Hib-II genomes are missing the liposaccharide-associated genes rffG, siaA, kfiC, orfE, wbaP/rfbP, and omp1P. The tryptophanase gene cluster is also present based on our mapping to NCTC8468 [30]. HI-EDI064 also seems to carry some genes associated with haemagglutinating pili [31]. Compared to the reference sequence, HI-EDI064 seemingly lacks the lex2 locus (no coverage in Lex2B and coverage in Lex2A is limited to a sequence of tandem repeats), which can be observed in Hia/Hid/Hif genomes. HI-EDI064 also lacks the non-pilus adhesin hmwABC but carries hap and hia [1]. As has been observed in NTHi genomes [1], the Hia-II and Hib-II genomes either lack or carry a highly divergent copy of the ompP1 gene, which is related to the infection of epithelial cells [32], present in all other capsular genomes. Finally, we investigated the presence of seven plasmids but could not find any evidence for them. We also investigated the presence and amino acid changes of 11 genes associated with antibiotic resistance (see Additional file 2: Table S12) and identified four PBP3 substitutions which have been associated with ampicillin resistance in the past (for a more extensive discussion, see Additional file 1).

### *Yersinia pestis* aDNA analysis (YP-EDI064)

While coverage from shotgun libraries was sufficient to reconstruct a *H. influenzae* genome, the *Y. pestis* data remained sparse. Consequently, we decided to enrich a full UDG-treated library for *Y. pestis* DNA using a custom in-solution target enrichment kit. After combining data of both the shotgun-sequenced non-UDG library and the captured full-UDG library, a chromosomal mean coverage of 0.9-fold was reached for YP-EDI064; the plasmids showed mean coverages of 2.6-fold (pCD1), 1.8-fold (pMT1), and 18.2-fold (pPCP1) (Additional file 2: Table S7 and Fig. S8). The coverage was sufficient to validate the presence of a *Y. pestis* infection, and damage plots generated from the mapping of the non-UDG data further support the authenticity of the aDNA sequences (Fig. S6). However, the chromosomal coverage, with only 53.5% of the sequence being covered at least 1-fold, is insufficient for reliable standard phylogenetic analyses.

Therefore, based on the assumption that EDI064 was infected with an identical or a similar strain as EDI001.A (SAMEA5661363; a high-coverage genome previously published for this site [14]), all shared and unique derived SNPs of available First Plague Pandemic genomes were manually assessed in IGV. Among the 45 derived SNPs shared between all first pandemic genomes, 25 were covered by at least one read (Additional file 2: Table S8). The otherwise identical YP-EDI064 and EDI001.A genomes differ in only two positions (1489055, 2352174). In both positions, YP-EDI064 has a mapping read with the ancestral state of the respective SNP (Fig. S7). Both divergences cannot be explained by deamination damage but might be due to erroneous mapping, potentially caused by conserved sequence intervals across closely related species. This is substantiated since the reads covering both positions carry an additional SNP in another position. Of the 39 phylogenetically diagnostic positions for the diversity within the first pandemic clade, i.e., not shared by all genomes, 22 were covered by at least one read (Additional file 2: Table S9), all of which were identical between YP-EDI064 and EDI001.A. Most notably, the unique EDI001.A SNP which forms the small branch between EDI001.A and the most recent common ancestor of all other European first pandemic genomes sequenced so far is covered by one read retrieved from the non-UDG shotgun library. Although a G>A SNP and therefore rescaled as potential damage, the frequency of G>A for this position (9th base on 3′) is estimated to be 1.4% based on MapDamage2 [33]. Assuming this SNP was correctly identified, the genome of YP-EDI064 would therefore be either identical with EDI001.A or could be derived from it.

### Human aDNA analysis

Human genome mappings were generated for both EDI064 and EDI086 at a depth of coverage of 0.61 and 1.0×, respectively. Fragment length and C>T misincorporation rates at the 5′ ends of reads indicate an authentic ancient source, and contamination rates based on mtDNA heterozygosity were low (0.47% and 0.63%, respectively). Both individuals are genetically male and have different mitochondrial haplotypes (H1a1 and K1c2), Y chromosome haplotypes (R1b-L11/L151 and R1b6-Z21/Z30), and a low estimated contamination rate based on heterozygosity of the X chromosome (< 1.1%). Using a pairwise comparison approach [34], neither male shows a close genetic kinship to the other, even though they were buried within the same grave. Due to the ultra-low coverage of our human genome mapping, genotypes could not be directly called in either individual. Variant positions listed in the latest version of the ClinVar database (17/06/2021) were called using ANGSD [35], by sampling a random read in pseudo-haploid calling mode. We filtered for pathogenic and likely pathogenic variation with no conflicting evidence and criteria provided by at least one submitter and checked our alignment for known variations that could potentially relate to *Y. pestis* and *H. influenzae* pathophysiology [36–38] (see the “Discussion” section); however, no risk factors were identified (see SI).

## Discussion

Plague has had an immense impact on European populations throughout its history, and its presence in the Anglo-Saxon cemetery of Edix Hill has been previously reported [14]. The case of the child EDI064 is the first instance of a co-infection with plague reported from the site. It confirms the presence of *H. influenzae* in non-adult populations as early as the sixth century CE and illustrates the toll plague took on populations, which were burdened by diverse pre-existing medical conditions, making them even more susceptible to epidemic diseases before the advent of modern medicine. Since commensal nasopharyngeal carriage would not be detectable in the bloodstream, which is a predicate for most non-dental ancient pathogen detection in teeth, we assume that the child was suffering from a systemic form of Hib infection. Additionally, osteological lesions indicative of invasive disease were recorded on the child’s bones.

The 6-year-old individual EDI064 exhibits lesions that can be associated with oligoarticular septic arthritis at the knees and possibly the ankles, which can result from an invasive Hib infection. Based on epidemiological and clinical studies, septic arthritis can be caused by a range of bacteria (of which no other was detected in EDI064), but in young children, the etiological agents have most frequently been identified as *H. influenzae* (pre-Hib vaccine) and *Staphylococcus aureus* (post-Hib vaccine) [39–41]. Septic arthritis can be mono- and oligoarticular, although in children, sites of infection are usually situated in the lower limbs (particularly knees, hips, and ankles). Most cases are reported for children between the ages of 1 month and 6 years, with a 2:1 ratio of boys to girls [39–41]. For Hib, septic arthritis in children occurs in about 8% of systemic cases [39]. It can be accompanied by a secondary inflammation such as meningitis or osteomyelitis and is usually preceded by an upper respiratory tract infection. The infection does not clear from the joint until successful treatment and was fatal in two-thirds of patients prior to the introduction of antibiotics, especially in cases of co-morbidities and oligoarticular infections [39, 42, 43].

EDI064 falls into the right demographic cohort for Hib-induced septic arthritis, although he is slightly older than average. Combined with the identification of Hib DNA in vascularized tooth roots, it seems likely that the osseous changes observed on the skeleton were indeed caused by septic arthritis. While we cannot exclude that the child also suffered from meningitis, our findings could not confirm this because of poor preservation of the endocranial surface. Children with untreated Hib bacteremia have been shown to develop more infection foci over time if left untreated [44]. Untreated septic arthritis often results in reduced and potentially impaired mobility [39, 41, 43], and if the child did in fact also suffer from untreated meningitis, neurological damage and hearing loss cannot be excluded [45, 46]. In children, untreated bacterial meningitis can lead to death in up to 5% of cases and to permanent disabilities in up to 40% of cases [5, 47].

While infections such as Hib can leave traces on the human skeleton, these lesions are generally indistinct. While they hint at invasive disease, it is nearly impossible to determine which condition caused the osseous changes without a molecular identification of the causative agent(s). Septic arthritis, for example, can point towards invasive infections from a multitude of bacteria, viruses, and even fungi, but the most commonly isolated pathogens are *H. influenzae*, *S. aureus*, *Streptococcus pneumoniae*, or *Kingella kingae*. Interestingly, the pathogens most likely to have caused illness can often change based on the age of the affected individual. For example, in historical populations, non-adults under the age of six with signs of septic arthritis and/or a meningeal infection are likely to have suffered from a Hib infection [40, 43]. While not a “clear” sign for either *H. influenzae* or other pathogens, these pathologies still point to invasive disease and hematogenous dissemination, which is ideal for pathogen detection in vascularized hard tissues such as teeth. This study illustrates a case that could inform us on more targeted sampling strategies for the study of Hib and other invasive respiratory pathogens causing meningitis, osteomyelitis, and septic arthritis, particularly in children.

Our virulence analysis showed that HI-EDI064 has a different virulence profile to most other Hib genomes, particularly all genomes belonging to the Hib-I clade (see Fig. 4 and Fig. S4). Based on the presence of the haemagglutinating pili gene cluster hifABCDE, the Anglo-Saxon strain was possibly fimbriated, i.e., long hair-like pili or fimbriae extended from its cell surface. The genes hifBC are known to be highly conserved in the species, while hifADE show higher sequence diversity [30, 48]. Our analysis showed significant coverage within the intervals for hifBCD. However, only 56.8% of hifE was covered (spread across the reference), and for hifA, mapping was restricted to 16.9% of the gene interval. Additionally, the mean edit distance for hifAE was markedly increased (mean above 2); hifAE are essential for the synthesis of mature pili that require the presence of all five hif genes [31, 49]. While hifE is probably present, the case of hifA is not as clear. Our results could either point to the lack of hifA in HI-EDI064 or the presence of a highly divergent copy of the gene. Previous studies seem to indicate that the probability of the presence of hifBC in *H. influenzae* without a full hif gene cluster is low, but it has also been stated that *H. influenzae* strains can use the presence/absence of the hif gene cluster as an adaptation mechanism [7, 50–52]. Fimbriated strains have been shown to adhere to mammalian extracellular matrices (ECMs), which could play a role in enhancing the pathogen’s effectiveness during bloodstream invasion. However, the expression of haemagglutinating pili in *H. influenzae* is subject to reversible phase variation, and fimbriated strains have been shown to preferentially express pili during nasopharyngeal colonization and upper respiratory tract infections rather than during bloodstream dissemination. It is therefore assumed that the main advantages conferred to fimbriated *H. influenzae* strains involve the adherence of nasopharyngeal and oropharyngeal epithelial cells during the initial phases of infection [7, 50–52].

Contrary to NCTC8468, HI-EDI064 does not seem to carry the lex2 locus, which is part of the lipopolysaccharide phase variation apparatus and is therefore involved in host environment adaptation and immune evasion [53]. Generally, we can observe that the virulence profile of HI-EDI064 in lipooligosaccharide-associated intervals more closely matches the profiles of Hia and Hic-f, which are all lacking genes found in Hib-I strains.

HI-EDI064 carries the sodC gene, which is only present in encapsulated *H. influenzae* strains grouping in phylogenetic division II. However, while *H. haemolyticus* and *H. parainfluenzae* carry an active sodC gene, *H. influenzae* genomes are only known to carry sodC as a pseudogene. The [Cu,Zn]-SOD protein lacks enzymatic activity, which has been associated with a point mutation (C>T, His>Tyr) in the enzyme active site for NCTC8468 [25, 54]. For HI-EDI064, the SNP is only covered once but carries the allele of the inactive gene and is unlikely to be caused by deamination based on its read position.

Although phylogenetic division II strains, which are mostly composed of Hie-f and NTHi strains, have been shown to be less likely to cause invasive disease than phylogenetic division I strains in the past [55], both Hib-II strains can be associated with invasive disease. The main virulence factor remains the expression of a capsule, particularly the serotype b capsule, which promotes intravascular survival and replication and protects the pathogen against phagocytosis and opsonization. It is by far the most defining factor when evaluating the pathogenic potential of an *H. influenzae* strain and seems to remain the defining factor of pathogenicity in Hib-II [55, 56].

The phylogenetic division of *H. influenzae* is estimated to predate human migration out of Africa and can be observed in gene-based, MLST-based, and full genome-based phylogenies alike [1, 3, 29]. Only serotypes a and b are represented in both divisions (see Fig. 3) [29], and it is hypothesized that an ancestral non-encapsulated strain acquired the serotype b capsule through recombination. However, the directionality or timing of this exchange remains unclear [29, 55]. Our ancient genome, HI-EDI064, clusters in phylogenetic division II and is most closely related to strain NCTC8468, which is also a Hib-II genome. While the division lacks a large number of Hia and Hib genomes, our phylogeny seems to point to a serotype-specific organization of phylogenetic division II. The similarity of HI-EDI064 to NCTC8468 which was isolated in 1940 could also point to a relatively clonal evolution of the Hib-II clade. Kroll et al. [25] suggest that division II is older than division I and that the presence of sodC in the former can be attributed to the genomic remains of an unencapsulated ancestral strain. Considering our data, which opens up the possibility for larger unsampled Hib-II diversity in the past, three scenarios to explain the presence of serotype b strains in both superclades are possible: (1) a non-encapsulated division II strains acquired the serotype b capsule from Hib-I, (2) the capsule was acquired by Hib-I via recombination from Hib-II, and (3) a capsule switch event led to the horizontal gene transfer of a Hib-I cap-locus region into a Hia-II strain. While the first scenario would have occurred at least once before the sixth century CE based on our strain, the timing of the second would have to be validated by further data. At the current stage of data collection, the basal position of our genome and the presence of sodC across phylogenetic division II do seem to point towards Hib-II predating Hib-I. However, the closeness of the Hib-II and Hia-II clades both in our phylogenetic and virulence factor analysis as well as our observation that the best hit for the cap locus region III gene hcsB most closely matched the profile of Hia-II genes for HI-EDI064 during serotyping (see methods) could also point to a capsular switch event, which has previously been described for capsular *H. influenzae* stains [3, 57]. While more ancient genomes will be needed to answer this question with certainty, our data demonstrates the potential of ancient genomes to elucidate the evolutionary history of Hib.

In addition to the identification of *H. influenzae*, we also identified *Y. pestis* aDNA in the teeth of EDI064. The non-adult plague victim was laid to rest in a double burial with a second unrelated adult putative plague victim. This underlines the value of multiple burials as indicators for past epidemic outbreaks and their suitability as targets for paleogenomic studies. Although the chromosomal coverage was too low for conventional phylogenetic methods, manual investigation of diagnostic SNPs revealed that the genome is likely identical or a descendant of the genome retrieved from individual EDI001 of the same site [14]. An association with the Justinianic plague in the 540s CE is further supported through radiocarbon dating (see Additional file 1).

Co-infections of modern *Y. pestis* strains with other pathogens have rarely been reported in the scientific literature, but recently, a case of co-infection with *Treponema pallidum pertenue* was reported for a plague victim in fifteenth-century Vilnius [13]. Today, *Y. pestis* outbreaks are more restricted to regions with limited medical capacity for large-scale testing; resources might even be more limited in cases of epidemics [58]. Moreover, the rapid treatment of suspected or confirmed *Y. pestis* infections with streptomycin likely also suppresses pre-existing or acquired co-infections of a broad range of pathogenic bacteria (including *H. influenzae*; cf. [59]) and therefore impedes their diagnosis. This is not the case for aDNA samples stemming from victims of bacterial infection prior to the introduction of antibiotics.

In this context, we investigated the potential effects of a secondary *Y. pestis* infection (for a more extensive discussion, see Additional file 1). In experiments, *Y. pestis* has been shown to be able to establish a permissive environment for the proliferation of other pathogenic bacteria [60]. Therefore, plague infections could act as enhancers for pre-existing or opportunistic bacterial infections. In conclusion, while the rapid progression of plague infections and the severity of the osseous changes observed on the skeleton of EDI064, which are consistent with prolonged illness and multiple infection foci, suggest that the *H. influenzae* infection was a pre-existing condition, a mechanism such as the pla-mediated inactivation of PAI-1 through *Y. pestis* could have further contributed to the progression of the *H. influenzae* infection through interference with the innate immune response and might have led to the rapid perimortem spread of Hib to additional infection foci.

Our results demonstrate the potential of aDNA data to inform us on plague co-infections and the role of plague as a co-infectant. Paleogenetically identified plague victims of past pandemics might allow for the observation of otherwise undetectable co-infecting pathogens. This underlines the importance of rigorous metagenomic screenings for identified plague victims.

Lastly, the differences in aDNA preservation for *Y. pestis* (GC% ~ 47) and *Hib* (GC% ~ 30–38), both facultative anaerobic bacteria, are striking. Even though *Y. pestis* genomes are more than double the size of a *H. influenzae* genome, the latter could be recovered almost completely while the former could not, even while enriching the library for *Y. pestis* DNA. Deamination rates seem to remain comparable. This disparity hints at the significant role played by perimortem bacteremia levels and clinical phenotypes in the recovery of pathogenic aDNA even under identical taphonomic conditions.

## Conclusions

From a paleogenomic perspective, the case of EDI064 highlights the suitability of aDNA metagenomic datasets for effective multi-pathogen detection and the importance of clinical phenotypes for pathogen aDNA recovery. Additionally, a synthesis of osteological and genomic analyses enabled the reconstruction of the genome of a bacterial pathogen often restricted to respiratory tract infections and the etiopathology of osteological lesions associated with it. With this, our study expands on available knowledge on childhood health prior to the introduction of modern medicine and illustrates the effect of the plague on already disease-stricken populations and immunocompromised children in particular. It also confirms that Hib was circulating in sixth-century Europe, probably with similar clinical features. Furthermore, our results point to the importance of *Y. pestis* as a co-infectant for the study of ancient pathogens.

While we are only adding one genome to the *H. influenzae* phylogeny, our analysis clearly illustrates the potential of ancient DNA to study *H. influenzae* and to get insights into the early evolutionary history of its most pathogenic serotype. The presence of a sixth-century strain in Hib-II carrying sodC opens the question of a Hib-II origin of the b-capsule and strengthens the argument of a phylogenetically older division II superclade, although more data is needed to fully answer these questions and capsule switch events cannot be excluded. Additionally, we can see that our Anglo-Saxon genome is genomically similar to another Hib-II genome of the 1940s, from which we conclude that evolutionary dynamics of this clade probably remained mostly clonal since the sixth century CE. The largely clonal organization of serotypeable strains makes the recovery of temporal signals or geographical structure for *H. influenzae* difficult. Accordingly, aDNA genomes are of particular interest as the addition of more data could help us elucidate these evolutionary questions. Further sampling will also be needed to explore the overall genetic diversity and prevalence of the species and the Hib-II clade in ancient populations. Genomes from different periods of human history could offer us a unique opportunity to study the evolution of the pathogen’s virulence and give us further insights into the expansion and rise of non-typeable strains, which have recently been emerging as the main cause of invasive *H. influenzae* infections [1, 6, 47].

## Methods

### Laboratory workflow

#### Sampling, extraction, and library preparation for NGS

Inside a class IIB hood in the dedicated aDNA facility of the University of Tartu Ancient DNA laboratory, the root portions of the teeth were removed with a sterile drill wheel. The root portions were briefly brushed to remove the surface dirt with full-strength household bleach (6% w/v NaOCl) using a disposable toothbrush that was soaked in 6% (w/v) bleach prior to use. Each root portion was then soaked in 6% (w/v) bleach for 5 min. The samples were rinsed twice with 18.2 MΩcm H2O and soaked in 70% (v/v) ethanol for 2 min, transferred to a clean paper towel on a rack inside a class IIB hood with the UV light on, and allowed to dry. They were weighed and transferred to PCR-clean 5-ml conical tube (Eppendorf) for chemical extraction. Inside a class IIB hood, 2 ml of 0.5 M EDTA Buffer pH 8.0 (Fluka) and 50 μl of Proteinase K 18.2 mg/ml (Roche) were added to each sample as well as a blank. The tubes were rocked in an incubator for 72 h at room temperature. The extracts were concentrated to 250 μl using Amplicon Ultra-15 concentrators with a 30-kDa filter (Millipore).

The extracts were purified according to the manufacturer’s instructions using buffers from the Minelute PCR Purification Kit (Qiagen) with the following changes: (1) the use of high-volume spin columns (Roche), (2) 10× PB buffer instead of 5×, and (3) samples incubated with EB buffer (Qiagen) at 37 °C for 10 min prior to elution. The columns were transferred to clean, labeled, 1.5-ml Eppendorf tubes. One hundred microlitres EB buffer is added to the membrane and centrifuged at 13,000 rpm for 2 min after the 10-min incubation and stored at−20 °C. Only one extraction was performed per sample for screening, and 30 μl was used for libraries.

Library preparation was conducted using a protocol modified from the manufacturer’s instructions included in the NEBNext® Library Preparation Kit for 454 (E6070S, New England Biolabs, Ipswich, MA) as detailed in [61]. DNA was not fragmented, reactions were scaled to half volume, and adaptors were made and used in a final concentration of 2.5 μM each. DNA was purified on MinElute columns (Qiagen). Libraries were amplified using the following PCR set up: 50 μl DNA library, 1× PCR buffer, 2.5 mM MgCl2, 1 mg/ml BSA, 0.2 μM Universal Primer (New England Biolabs), 0.2 mM dNTP each, 0.1 U/μl HGS Taq Diamond (Eurogentec), and 0.2 μM indexing primer. Cycling conditions were 5′ at 94 °C, followed by 18 cycles of 30 s each at 94 °C, 60 °C, and 68 °C, with a final extension of 7 min at 72 °C. Amplified products were purified using MinElute columns and eluted in 35 μl EB (Qiagen). Three verification steps were implemented to make sure library preparation was successful and to measure the concentration of dsDNA/sequencing libraries—fluorometric quantitation (Qubit, Thermo Fisher Scientific), parallel capillary electrophoresis (Fragment Analyser, Advanced Analytical), and qPCR.

#### *Yersinia pestis* target enrichment

Due to the low coverage of our initial shotgun *Y. pestis* mapping, we enriched the shotgun library of sample EDI064 for *Y. pestis* DNA using an Arbor Biosciences Custom MyBaits *Y. pestis* capture kit (v4) that includes baits covering the genomes for *Y. pestis* CO92 (including all plasmids) and *Y. pseudotuberculosis*. The captured library was amplified using 2× KAPA HiFi HotStart ReadyMix DNA Polymerase and primers IS5 and IS6 [61]. Following amplification, the library was sequenced on a NextSeq500 platform (MID150, PE) at the University of Tartu, Institute of Genomics Core Facility, with other capture samples.

### Bioinformatic workflow

#### Data preparation

Two libraries were sequenced on an Illumina NextSeq500 (75 bp, SE) at the DNA Sequencing Facility in the Department of Biochemistry, University of Cambridge, and on an Illumina NextSeq500 (75 bp, SE) at the University of Tartu, Institute of Genomics Core Facility. Two runs were sequenced per library. Sequencing data quality was ascertained using FastQC [62] at multiple stages and compared using MultiQC [63]. Raw sequencing datasets were trimmed and filtered using cutadapt [64] ( -m 30 --nextseq-trim=20 --times 3 -e 0.2 --trim-n) and deduplicated using ParDRe [65]. Raw data filtering for our *Y. pestis* analysis is described below.

#### Metagenomics

Deduplicated files were merged by sequencing run and analyzed using Kraken2 [22]. We used a custom database containing all dusted bacterial, viral, archaeal, and protozoan reference sequences, as well as the UniVec_Core database. Kraken2 output files were filtered for 152 pathogenic taxa. Additionally, we computed the metagenomic profile for our sample using KrakenUniq [23]. Here, the custom database contained dusted complete genomes and chromosome-level assemblies of bacteria, viruses, archaea, and protozoa. Additionally, this database contained the human genome, the NCBI Viral Neighbor database, and the contaminant databases UniVec and EmVec. The KrakenUniq algorithm is better suited to identify low abundance hits and is able to identify unique *k*mers as well as ascertain the duplication rate and coverage of the *k*mer dictionaries of each taxon. To take advantage of this, we computed a heatmap using plotly, pandas, matplotlib [66], and numpy [67] based on an *E*-value calculated as follows: $\left(\frac{K}{R}\right)\times C$. Here, *K* is the *k*mer count, *R* is the read count, and *C* is the coverage of the taxon *k*mer dictionary. The *E*-value cutoff for further inspection was 0.001.

#### *Haemophilus influenzae* analysis

i. *Haemophilus influenzae* mapping

- *Ancient data:* Data were merged by library and mapped against the *Haemophilus influenzae* NCTC8468 (GCF_901472485.1) reference sequence using bwa aln (-n 0.1 -l 1000) and bwa samse [68]. Alignments were then converted, sorted, and indexed using samtools [69]. We used Picard’s MarkDuplicates module to remove all duplicates. Libraries were then merged using samtools, and reads were realigned around indels using the GATK modules RealignerTargetCreator and IndelRealigner [70]. Finally, deamination profiles were computed, and read quality was rescaled based on damage patterns using mapDamage2.2.1 [33] and edit distance was computed with ED-NM_CSV [71].
  The quality of the mapping was assessed with Qualimap [72] and with aDNA-BAMPlotter [73] to visualize the alignment, deamination curves, and edit distance profiles.

b) *Modern data:*

*Assemblies, scaffolds, and contigs:* Chromosome, scaffold, and contig level data were downloaded from NCBI and fragmented with FASTA-FRAG [74]. The sequences were cut in 50-bp fragments tiled across the whole sequence 20× with two base pair intervals. Data were then mapped to the *H. influenzae* NCTC8468 (GCF_901472485.1) reference sequence using the same bwa, samtools, and Picard settings as detailed above for ancient DNA.

*SRA data:* SRA data were trimmed and quality filtered using cutadapt (as described above) and merged with FLASH [75] (-z -M 125). Data were then mapped to the *H. influenzae* NCTC8468 (GCF_901472485.1) reference sequence using the same bwa, samtools, and Picard settings as detailed above for ancient DNA. Raw Illumina paired-end reads of serotypeable datasets published in Potts et al. [29] were mapped without merging and using bwa paired-end mode (aln/sampe) instead.

ii. Comparative mappings

Ancient data were mapped non-competitively to reference sequences for *Haemophilus aegyptius* (GCF_900475885.1), *Haemophilus parainfluenzae* (GCF_000210895.1), and *Haemophilus haemolyticus* (GCF_003352385.1) using the same workflow as described for the *H. influenzae* NCTC8468 above. Additionally, the EDI064 data was mapped non-competitively to multiple serotype references within the *H. influenzae* species due to the high genetic diversity of the species (47% core genome based on parsnps [76] alignment) (representative genomes for serotypes a–f: CP017811.1, GCF_000210875.1, GCF_003351605.1, NC_000907.1, GCF_900478735.1, GCF_000465255.1). The highest sequence coverage and depth were achieved for the reference sequence for serotype f (KR494), which in combination with the presence of gene sodC was an indication for a phylogenetic division II strain like the final reference sequence NCTC8468.

iii. MLST

Multilocus sequence typing was done using the program mlst [77] and the integrated PubMLST *H. influenzae* typing scheme. For SRA data, reads were first assembled to contigs and scaffolds using SPAdes [78]. Typing was mostly performed using scaffolds. In a few cases, scaffolds were of poor quality, and contigs were used for typing instead. For our ancient genome, we called consensus sequences of the gene intervals needed for typing using Geneious Prime [79].

iv. Serotyping

In order to determine the serotype of the ancient *H. influenzae* strain, we used fasta files from hicap [3] and Potts et al. [29] “hinfluenzae_capsule_characterization” typing databases. Due to the lack of full assembly for our ancient sample, we built a multi-fasta out of all sequences and mapped it to our raw data using bwa mem with the -a flag to output all alignments as the database contains a number of sequences with a high percentage of identity, including multiple alleles of the same gene intervals. We then converted our alignment to a sorted BAM using samtools [69] and computed the sequence coverage of each interval using bedtools [80]. The reference multi-fasta was analyzed with prinseq [81] and GenMap [82] to check for sequence complexity and mappability. For the heatmap, we used the maximum coverage for each gene among Potts et al.’s sequences. Interestingly, we found that a cap locus region III gene, hcsB, which is common to all capsular strains irrespective of serotypes, showed better coverage within alleles most closely matching Hia strains for the aDNA strain HI-EDI064. NCBI datasets were directly serotyped using the hicap and hinfluenzae_capsule_characterization programs. Samples were used if a clear serotype could be assigned, i.e., if the data was not contaminated and there were no missing or truncated genes (exceptions are M14416, M13539, M21328 for which results were uncertain). NTHi strains from Potts et al. [29] were not used.

v. Mappability

Mappability across the reference genome was calculated with GenMap [82] with the settings -K 30 -E 2.

vi. Virulence screening, gene presence-absence, and plasmids screening

We built a database of virulence-associated intervals in the *H. influenzae* species based on the Virulence Factor Database (VFDB) [83] and Pinto et al. [1]. The mappability of the database was assessed using GenMap (-K 30, -E2) and its GC content and sequence complexity using a sliding window analysis. We then mapped all encapsulated genomes represented in our phylogeny to these intervals using bwa aln (-n 0.01 -l 1000). Coverage statistics for each interval were extracted using bedtools. The generated data were compiled and plotted in a clustermap and a multidimensional scaling plot using seaborn, pandas, numpy, scipy, sklearn [84], and matplotlib in python 3 using cosine distance.

Additionally, we built references for known *H. influenzae* plasmids, for which reference sequences were available, and genes associated with antibiotic resistance for *H. influenzae*. We mapped our data competitively to the references using bwa (-n 0.01 -l 1000). The plasmid reference includes seven full references (pA1209/NC_019185.1, pLFH49/NC_019184.1, pLFS5/NC_019183.1, pLFH64/NC_019182.1, pA1606/NC_019180.1, ICEhin1056/NC_011409.1, pJ612/NC_019186.1), and the antibiotic resistance genes reference panel includes eleven gene intervals (CP005967.1:1556773-1555592, CP005967.1:1193031-1194470, NC_000907.1:1197840-1199672, CP002276.1:1501099-1500536, CP002276.1:1499308-1496210, CP002276.1:1500456-1499308, NC_019180.1:2400-1540, NG_049977.1, NG_048163.1, NG_048217.1, NC_011409.1:18211-17570). Coverage in the plasmid intervals was either zero or limited to one to nine reads. In conclusion, we could not detect any of the seven plasmids in our datasets.

vii. SNP call and initial alignment

SNPs for all 493 genomes were called using GATK 3.5 UnifiedGenotyper with the EMIT_ALL_SITES flag and downsampling disabled. For modern genomes which were aligned using full genome assemblies, the default base quality was set to 30. BAM files were filtered for reads above MQ30 before SNP calling. We used MultiVCFAnalyzer [85] to generate a full sequence alignment using the NCTC8468 reference sequence. Heterozygous and homozygous SNPs were called above a minimum genotype quality threshold of 30, provided the position was covered by at least three reads of which 90% or more supported the call (for HI-EDI064 66.59% of the sequence was covered > 3×).

viii. Intervals not included for SNP call

To account for recombinant sites (the phi test showed a high probability for recombination in our alignment with a *p*-value < 0.00001), we used Gubbins to filter out recombinant sites [86]. Due to the small core genome size in our alignments, the filter percentage was set to 60, with 10 iteration and fast-tree as the tree builder. We removed all repeats, mobile elements, prophage-associated intervals, tRNA/rRNA genes, and conserved intervals causing overtiling from the resulting SNP alignment. This was repeated for all phylogenetic division II genomes separately. The partial deletion was applied to both alignments (90% for the full alignment and 95% for the phylogenetic division II alignment). EDI064 SNPs in the final alignments were inspected by the eye to exclude potentially wrong calls caused by deamination or misalignments.

ix. Phylogenetic analysis

Using the filtered SNP alignments, we built maximum likelihood trees using IQ-TREE2 [28]. The modelfinder implemented in IQ-TREE2 was used to find the appropriate model for both alignments; model search was restricted to the general time reversible model (a total of 22 GTR models were tested).

For our full phylogeny including both phylogenetic divisions, we used a total of 493 genomes and a final SNP alignment of 4712 bp following partial deletion (90%). IQ-TREE2 chose the GTR+F+ASC+R3 model as the best fitting model for the input alignment, which also applies an ascertainment bias correction for SNP alignments. For our phylogenetic division II phylogeny, we used 259 genomes and 10,041 positions after partial deletion (95%). The best-fitting model was GTR+F+ASC. Branch support was assessed with 1000 standard bootstrap replicates.

x. SNP annotation

For SNP effect analysis, we called all SNPs from our HI-EDI064 mapping to NCTC8468 using samtools mpileup and filtered using bcftools (QUAL > 19, DP ≧ 2 and ALT > 80%). We ran SNPEff [87] with the NCBI RefSeq annotation for NCTC8468. SNPs with predicted HIGH effects were inspected manually.

xi. Circos

The genome coverage plot was generated using Circos [88]. Coverage, GC-Skew, and GC% were calculated in 1000-bp windows using GC-GCSkew_Calculator [89]. The second ring is a heatmap based on mappability values generated using GenMap (see above).

#### *Yersinia pestis* analysis

For our analysis of *Y. pestis*, raw sequencing data was processed with the pipeline nf-core/eager version 2.2.0dev and [90]. Adapter clipping was performed with AdapterRemoval v2.3.1 [91] in nf-core/eager standard settings, mapping was done with BWA v0.7.17-r1188 [68]. The shotgun sequencing data of the non-UDG library was initially mapped with the parameters -n 0.01 and -l 16. For both the chromosome and the plasmids, the reference sequences of CO92 were used ([92]; chromosome: NC_003143.1; pMT1: NC_003134.1; pCD1: NC_003131.1; pPCP1: NC_003132.1). However, the reference sequences for the plasmids were merged to perform a competitive mapping. Sequencing data of the full-UDG library enriched for *Y. pestis* was mapped with -0 0.1 and -l 32. Mapped reads were then converted, sorted, indexed, and filtered for mapping quality > 37 with Samtools v.1.9 [69]. DeDup v. 0.12.6 [93] was used for deduplication. To achieve the highest possible coverage, both datasets were merged. To accommodate for the damage in the shotgun non-UDG data, the mapped reads were rescaled with mapDamage v2.7 [33] in default settings and remapped with nf-core/eager, using samtools fastq to convert the BAM file and BWA for remapping with the parameters -n 0.1 and -l 32. Due to low coverage, genotyping and phylogenetic analyses were not attempted. However, diagnostic SNPs within the Justinianic plague clade [14] were investigated with IGV 2.8.13 [94].

#### Human DNA analysis

i. Alignment to the reference sequence and quality control

The sequence reads were mapped to the human reference sequence (GRCh37/hg19) using Burrows-Wheeler Aligner (BWA 0.7.12) [68] command aln with seeding disabled. After mapping, the sequences were converted to BAM format, and only sequences that mapped to the reference genome were kept using samtools 1.9 [69]. Next, multiple BAMs from the same individual but different runs were merged using samtools merge; reads with mapping quality under 30 were filtered out, and duplicates were removed with picard 2.12 (http://broadinstitute.github.io/picard/index.html).

ii. aDNA authentication

Samtools-1.9 [69] option stats and BAMstats-1.25 (http://bamstats.sourceforge.net/) were used to determine the number of final reads, average read length, average coverage, etc. As a result of degradation over time, aDNA can be distinguished from modern DNA by certain characteristics: short fragments and a high frequency of C>T substitutions at the 5′ ends of sequences due to cytosine deamination. The program mapDamage2 [33] was used to estimate the frequency of 5′ C>T transitions (Additional file 2: Table S10). To estimate the level of potential contamination in the libraries (Additional file 2: Table S10), we used the human genome in two ways, the first estimating mtDNA contamination was estimated using the method from [95], which aligns the raw mtDNA reads to the RSRS [96], determines the haplotype using GATK pileup [97], counts the number of heterozygous reads on haplotype-defining sites as well as adjacent sites, and calculates a ratio that takes into account ancient DNA damage by excluding positions where a major allele is C or G and the minor is T or A, respectively. The second estimation is performed using a similar calculation on the X chromosome as implemented in ANGSD [35], the output of which is reported in Additional file 2 Table S10.

iii. Genetic sex estimation

Genetic sex was calculated (Additional file 2: Table S10) using the script sexing.py from [98], estimating the fraction of reads with mapping quality > 30 mapping to the Y chromosome out of all reads mapping to either the X or Y chromosome. Genetic sexing confirmed morphological sex estimates.

iv. Determining mtDNA haplogroups

Raw reads were mapped to the revised Cambridge Reference Sequence [99], and the resulting BAM files were indexed using samtools-1.9 [69]. Variants were called using Samtools 1.9 mpileup variant-only option [69] and filtered using bcftools v 1.1 [69]. Haplogroups were assigned using Phylotree build 16 [100] accessed at www.phylotree.org and Haplogrep [101] accessed at https://haplogrep.uibk.ac.at. The results are reported in Additional file 2 Table S10.

v. Y chromosome variant calling and haplotyping

Y chromosome variants were called as haploid, picking one allele at random (--doHaploCall 1) in ANGSD-0.916 [35] and filtered for regions that uniquely map to Y chromosome, retaining 8.8 Mb, when using short-read sequencing technology [102]. Haplogroup assignments were made on the basis of in silico genotyping of the samples for 108,000 informative variants 1000 Genome Project populations [103] in 456 geographically diverse high-coverage Y chromosome sequences [102] and those annotated by https://isogg.org/tree/ and https://www.yfull.com/. In haplogroup labeling, we followed the nomenclature of Karmin et al. [102]. The results are reported in Additional file 2 Table S10.

vi. Variant calling

The latest ClinVar vcf file was downloaded on 19/06/2021 and sites file containing the chromosome, position, and major and minor allele was created from the vcf for the ANGSD software using awk and bcftools. BAM files were then called at the ClinVar SNP sites using ANGSD [35] with the --doHaploCall 1 and --doMajorMinor 3 options. Variants were converted to PLINK format and then to vcf using PLINK-1.9 [104] and annotated with bcftools [105].

## Supplementary Information

**Additional file 1.** Supplementary figures and text [108-142].

**Additional file 2.** Supplementary tables.

**Additional file 3.** Review history.

## Data Availability

Sequencing data is available at the European Nucleotide Archive (ENA) under the accession number PRJEB45013 [106].
