# Supplementary material for: An invasive Haemophilus influenzae serotype b infection in an Anglo-Saxon plague victim
Source: Genome Biol. 2022 Feb 3;23:22. doi: 10.1186/s13059-021-02580-z (PMC8812261; doi:10.1186/s13059-021-02580-z)
Supplement: Supplementary file 1 — Additional file 1. Supplementary figures and text [108-142]. [file 13059_2021_2580_MOESM1_ESM.pdf]

Supplementary Information

An invasive *Haemophilus influenzae serotype b* infection in an Anglo-Saxon juvenile Plague victim

Meriam Guellil, Marcel Keller, Jenna M. Dittmar, Sarah A. Inskip, Craig Cessford, Anu Solnik, Toomas Kivisild, Mait Metspalu, John E. Robb, Christiana L. Scheib

Table of content:

I. Supplementary Figures

II. Supplementary Text

1. The potential role of *Y. pestis* as enhancer for pre-existing or opportunistic bacterial infections (Marcel Keller)
2. Radiocarbon dating
3. SnpEff analysis
4. Eerkens et al. 2018 RN4 re-analysis
5. Antibiotic-resistance associated loci analysis

Note: References can be found in the main manuscript.

I. SUPPLEMENTARY FIGURES

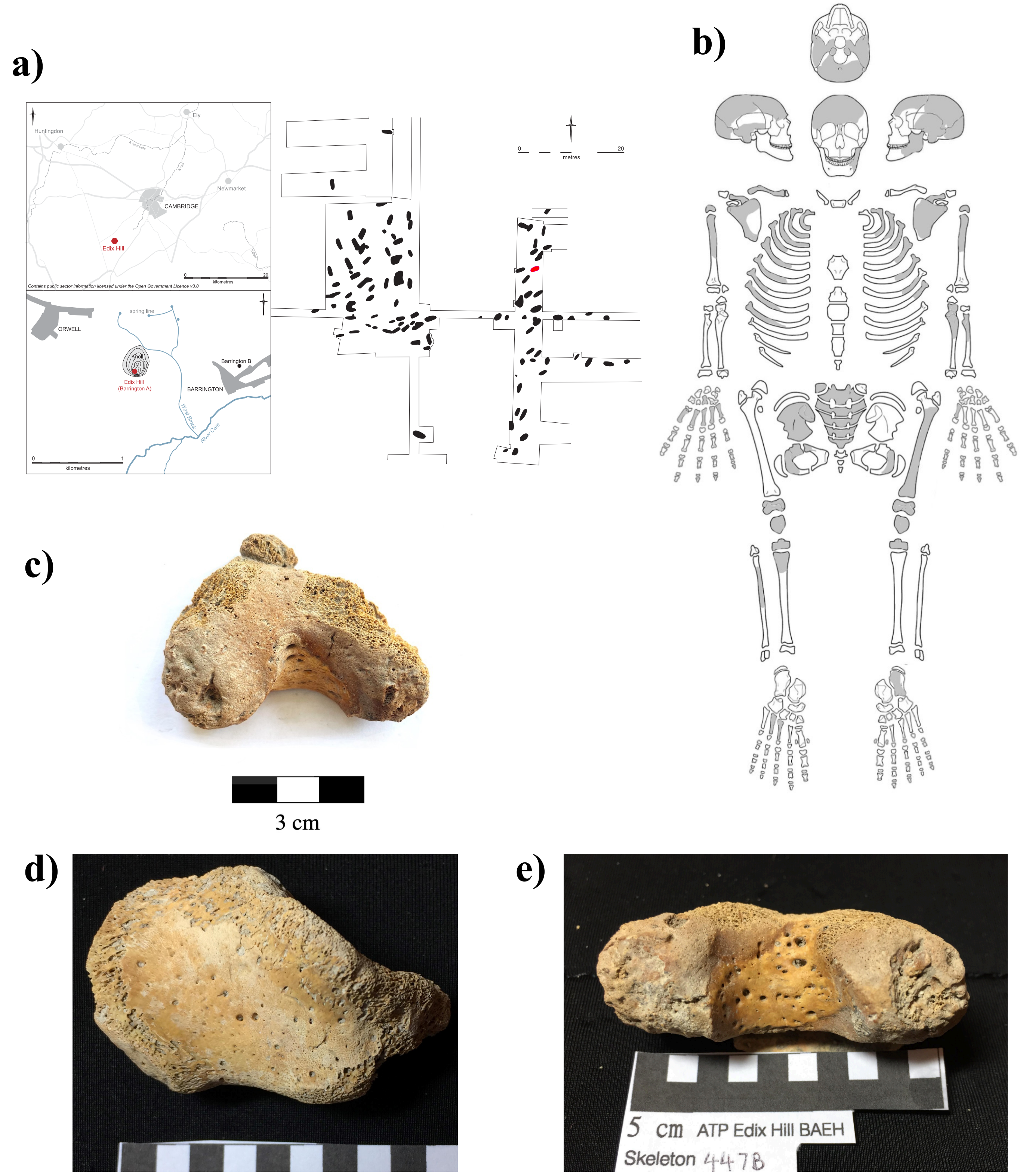

Figure S1: a) Plan of the Barrington A, Edix Hill, archaeological site with a map of the surrounding area (based on Malim and Hines 1998, various figures, redrawn by Vicki Herring for the After the Plague project). The grave from which EDI064 was excavated is marked in red. **b)** Skeletal record form for the juvenile individual EDI064. Graphical representation of the skeletal inventory (areas shaded in grey were not recovered from the grave). **c)** Right patella of EDI064 that is ankylosed to the anterosuperior aspect of the right distal femoral epiphysis (inferior view). Photograph by J. Dittmar. d) Image of the left calcaneus with region of periosteal new bone on the medial portion of the calcaneal tuberocity. Photograph by S. Inskip. e) Right distal femoral epiphysis with cortical defects. Photograph by S. Inskip.

a)

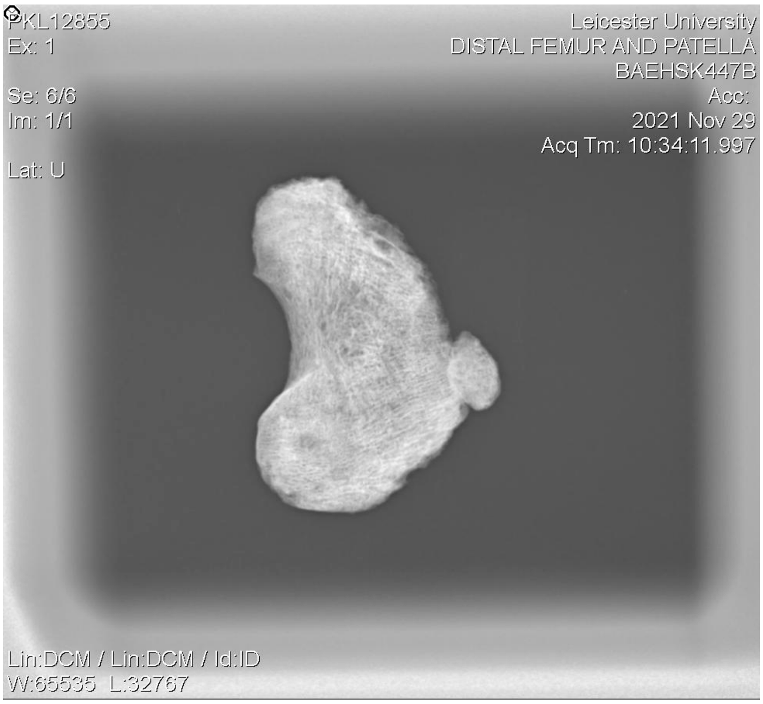

b)

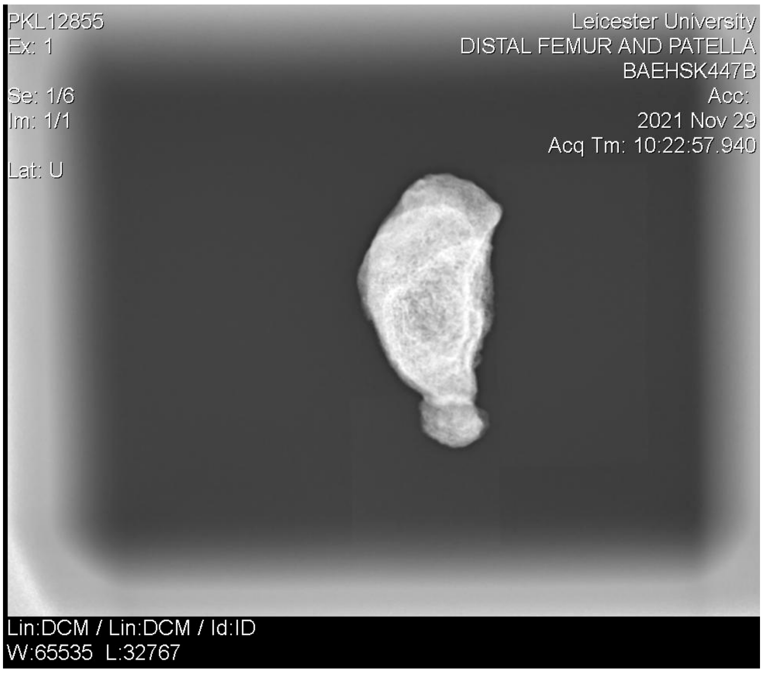

Figure S2: X-rays of the ankylosed right patella (Fig. S1c): a) Inferior view of the distal right epiphysis of the right femur with patella; b) Plain X-ray showing the lateral view of the distal epiphysis of the right femur with patella fused on to the anterior superior margin. X-rays taken by S. Inskip at Leicester University.

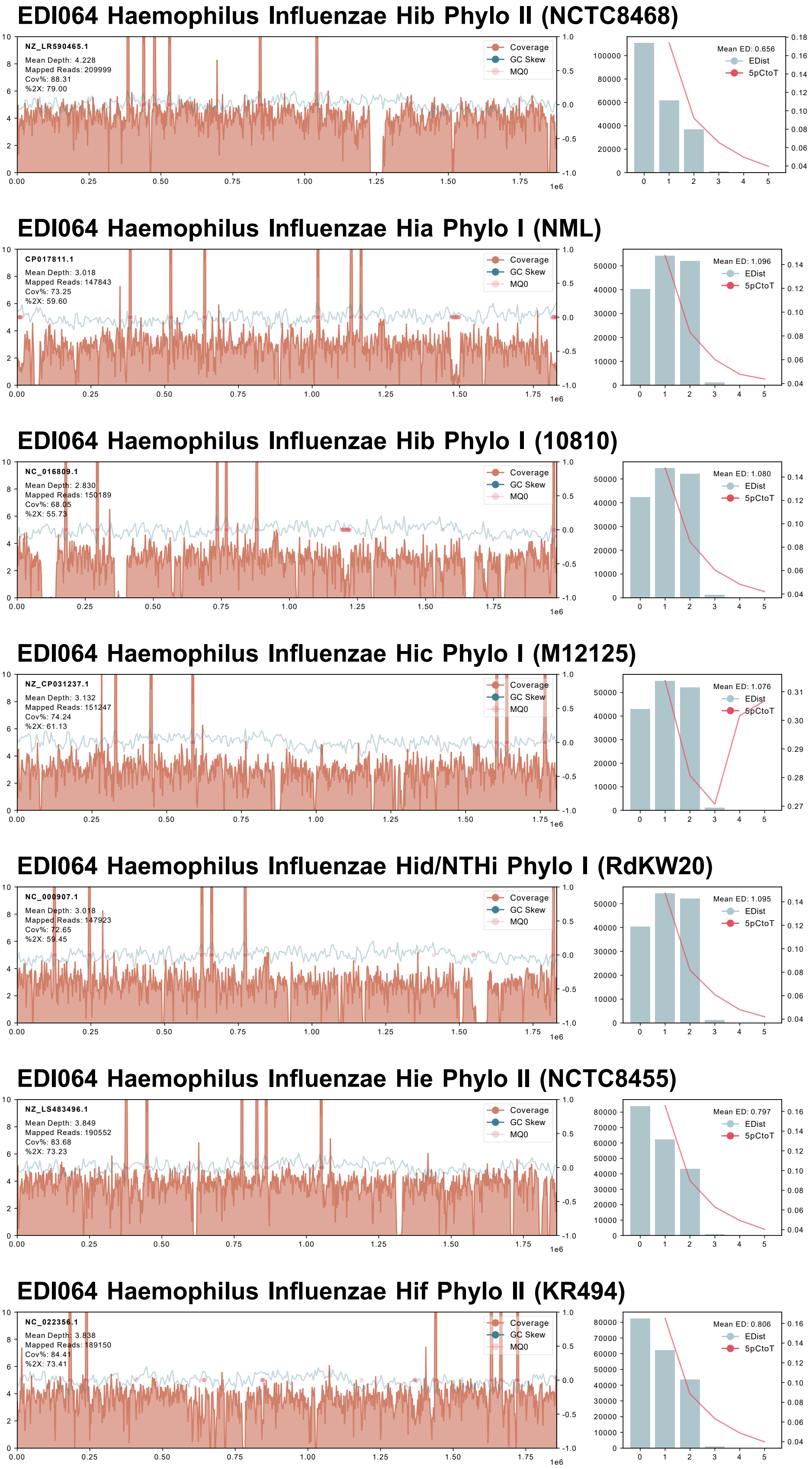

Figure S3: Non-competitive comparative mappings for the final reference sequence and reference sequences for each serotype (a-f). Plots on the left show coverage across the genome, plots on the right depict 5pCtoT deamination signatures (calculated using mapDamage2) and a histogram of read edit distances (-n0.1, MQ>0).

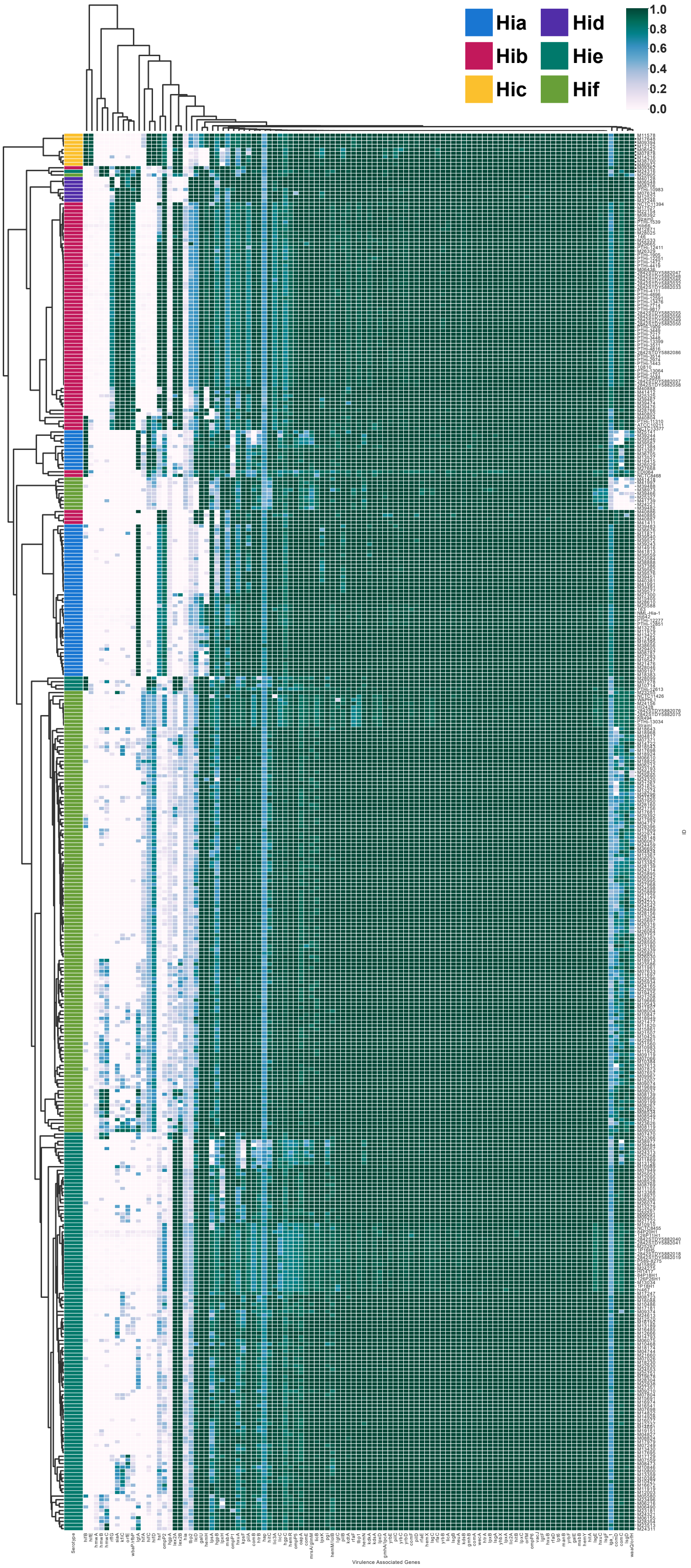

Figure S4: Full version of the clustermap presented in Fig. 3 in the main manuscript. Clusters for Hie and Hif are not collapsed. The clustermap has been generated based on coverage values in virulence-associated gene intervals for typable genomes using seaborn. Clusters were calculated based on cosine distance.

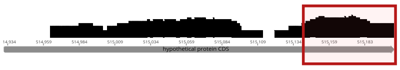

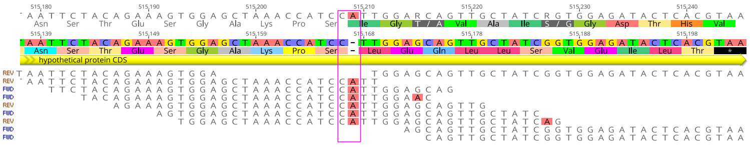

Gene: Hypothetical Protein
SNP: 515,167 (Insertion; C>CA)
Predicated Effect: frameshift variant

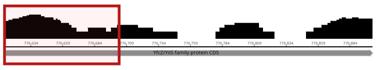

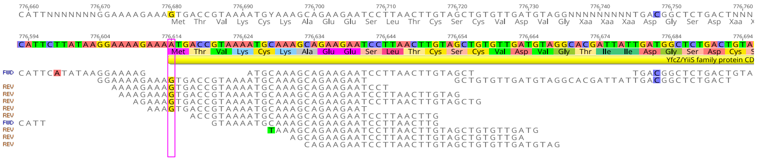

Gene: YfcZ/YiiS
SNP: 776,614 (A>G)
Predicated Effect: start lost

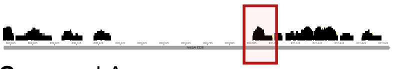

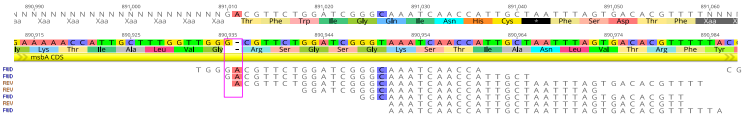

Gene: msbA
SNP: 890,935 (Insertion; G>GA)
Predicated Effect: frameshift variant

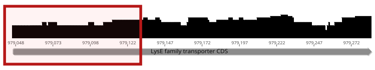

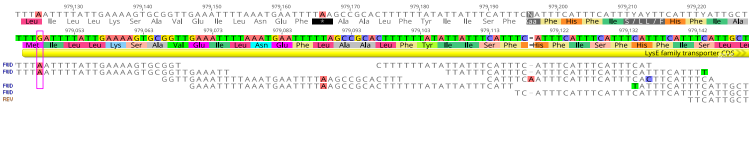

Gene: LysE
SNP: 979,089 (G>A)
Predicated Effect: stop gained

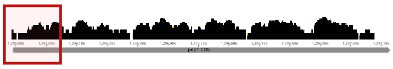

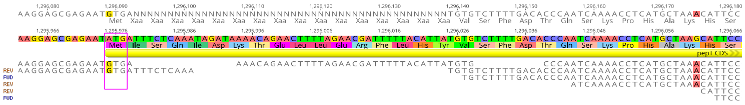

Gene: pepT
SNP: 1,295,975 (A>G)
Predicated Effect: start lost

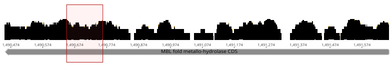

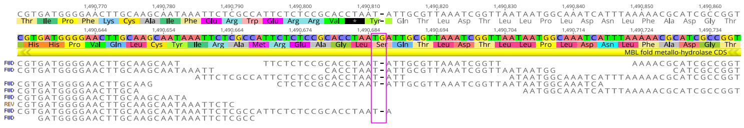

Gene: MBL fold metallo-hydrolase
SNP: 1,490,685 (Deletion; TG>T)
Predicated Effect: frameshift variant

Figure S5: HI-EDI064 SNPs with predicted high effect and coverage above 1X (Min DP2, 80%ALT allele) (SNPeff analysis on NCTC8468 mapping, MQ>30). Read alignments were visualised in Geneious Prime (2020.0.4) [79].

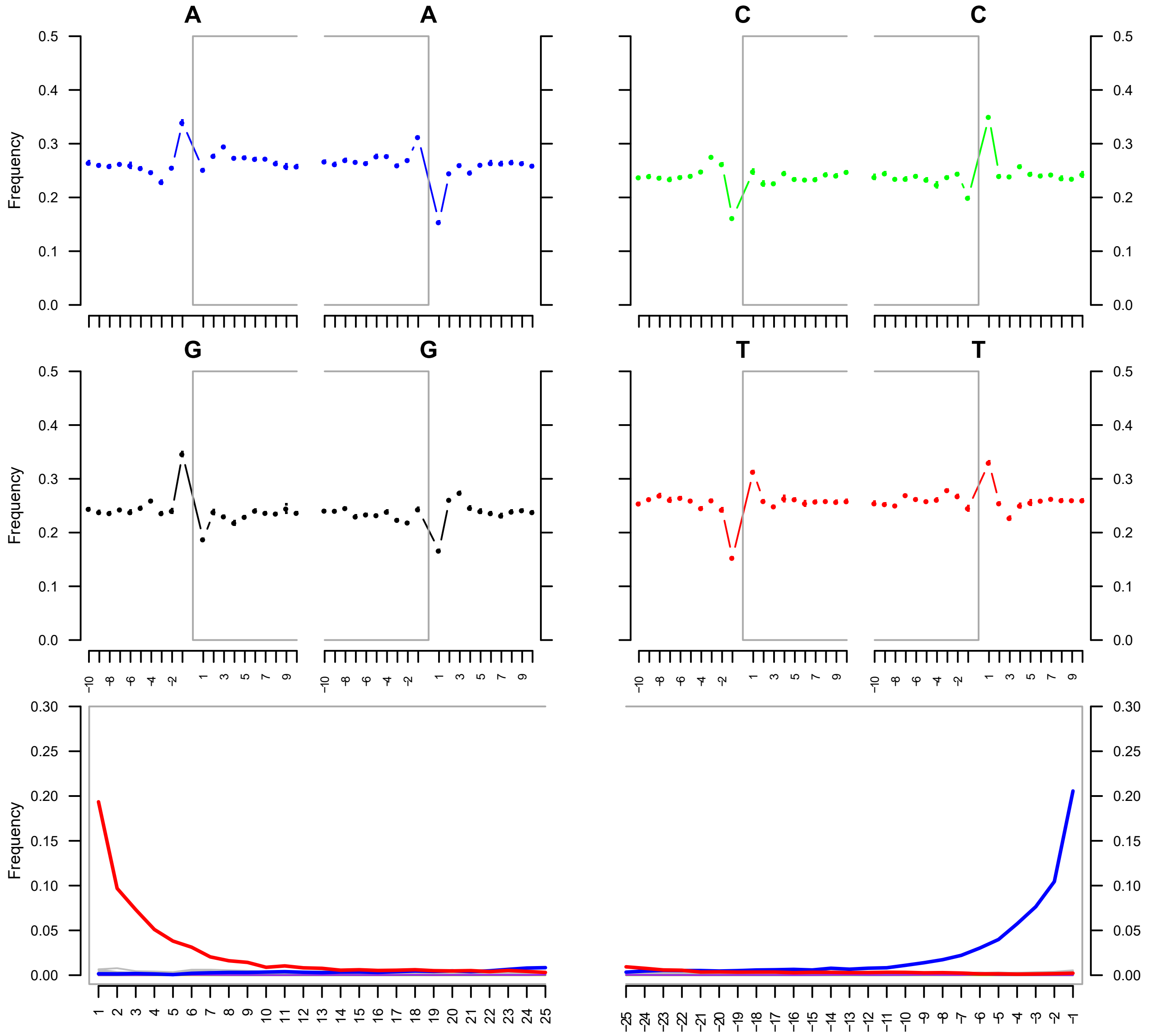

Figure S6: mapDamage2 plots of our mapping to the *Yersinia pestis* reference genome CO92 (-n 0.1; MQ>30) for the EDI064 sequencing data (nonUDG-shotgun and fullUDG-capture merged).

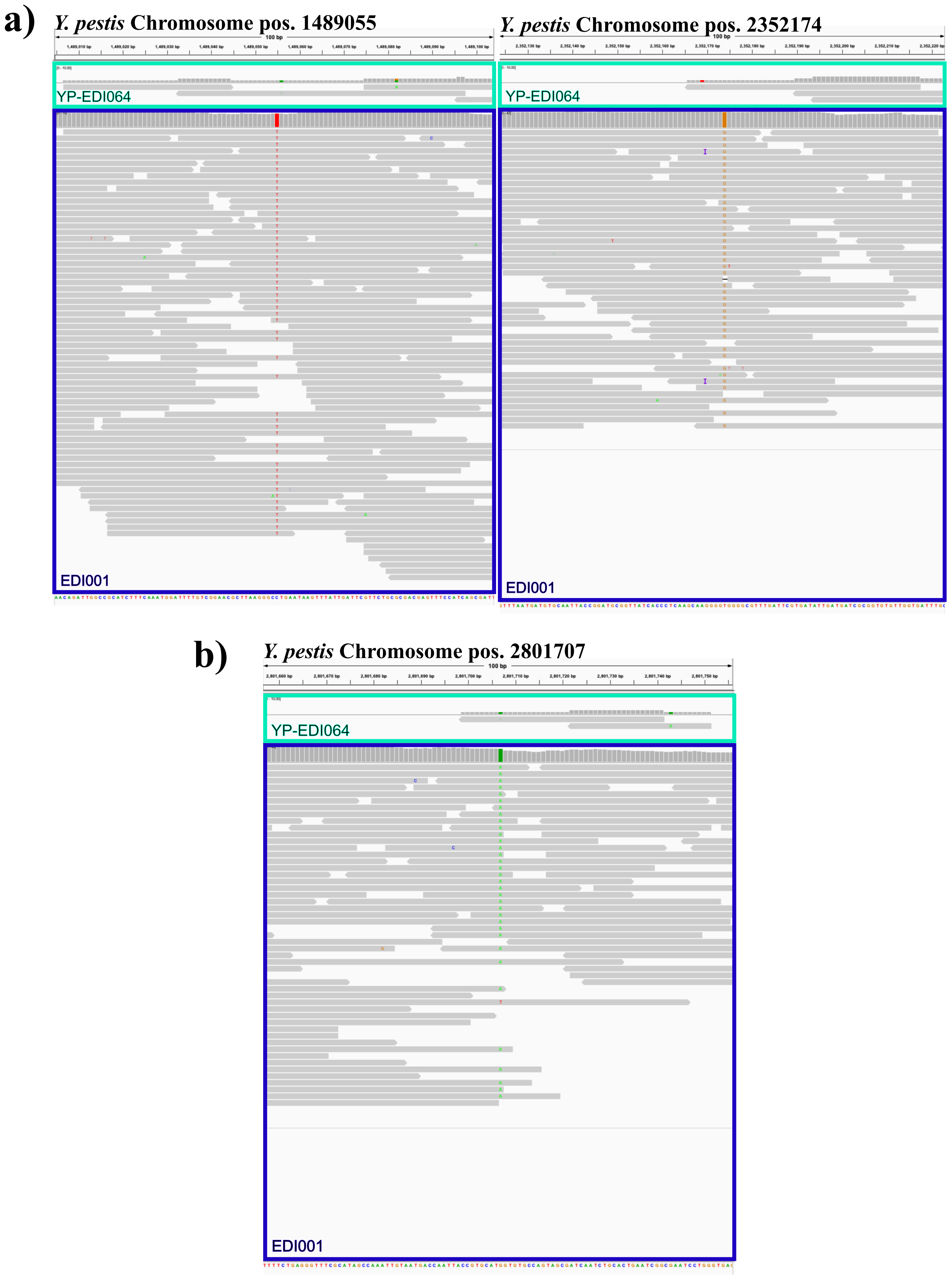

Figure S7: Screenshots of EDI064 (upper panel) and EDI001 (lower panel) reads mapped to the *Yersinia pestis* reference genome CO92 in IGV [142]: **a)** positions 1489055 and 2352174 for which EDI064 shows a reference call, potentially due to mapping of non-*Y. pestis* reads; **b)** position 2801707 with the unique SNP of EDI001, which defines its short branch, present in a single read in EDI064.

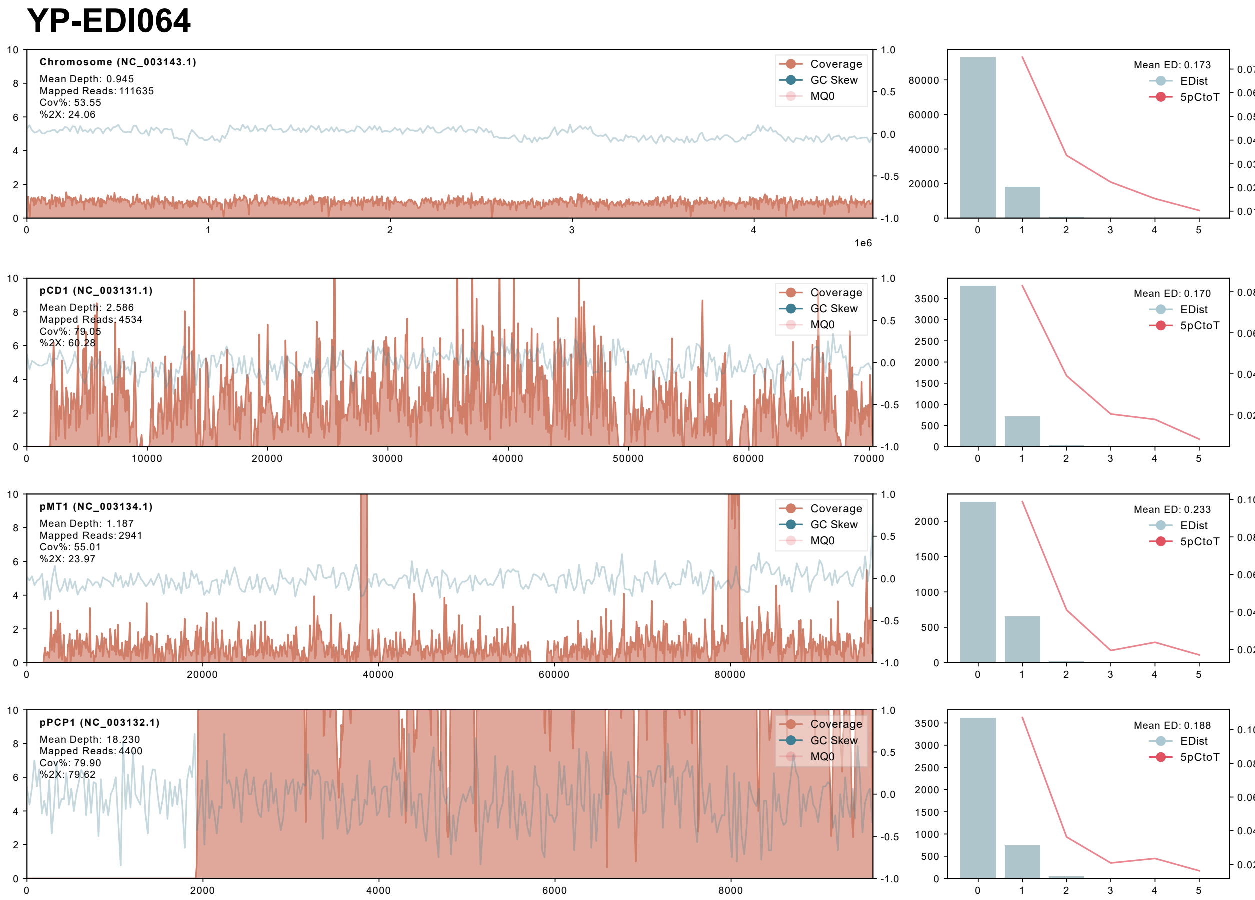

Figure S8: Coverage plots (left) and diagrams showing edit distance and damage pattern (right) for the chromosome, pCD1, pMT1 and pPCP1 using our EDI064 sequencing data (nonUDG-shotgun and fullUDG-capture merged). Data was mapped to the *Yersinia pestis* reference genome CO92 (-n 0.1; filter MQ>30 was applied, repeated section of the plasmids show no coverage). See Methods for details about data processing.

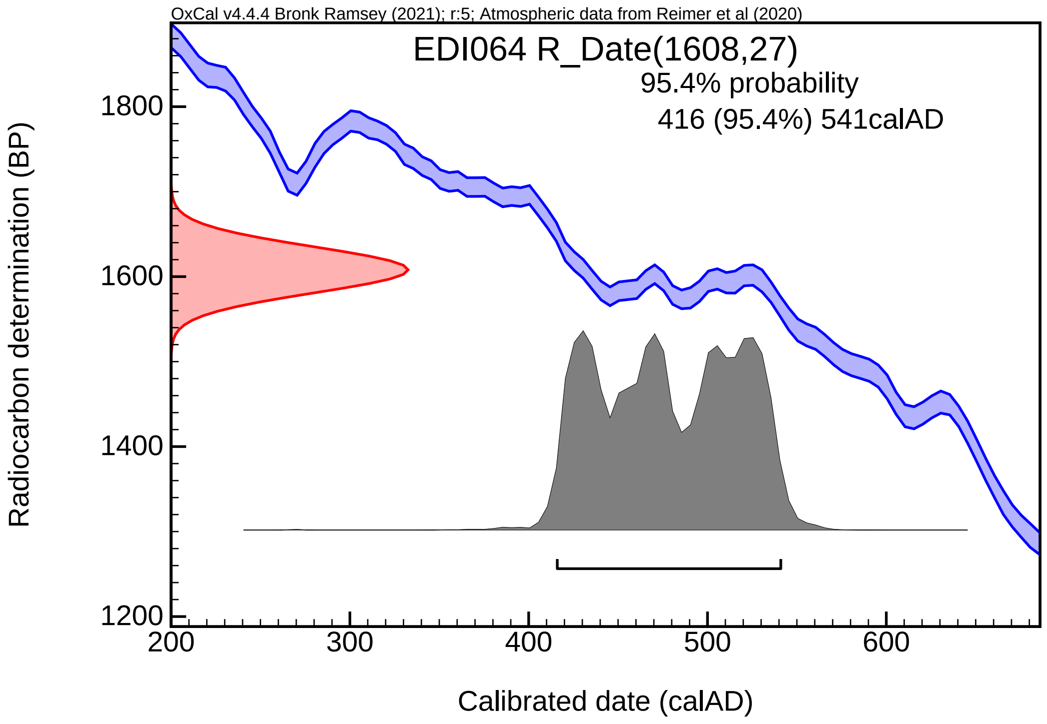

Figure S9: Radiocarbon date for the individual EDI064, calibrated with IntCal20 in OxCal v4.4.4.

a)

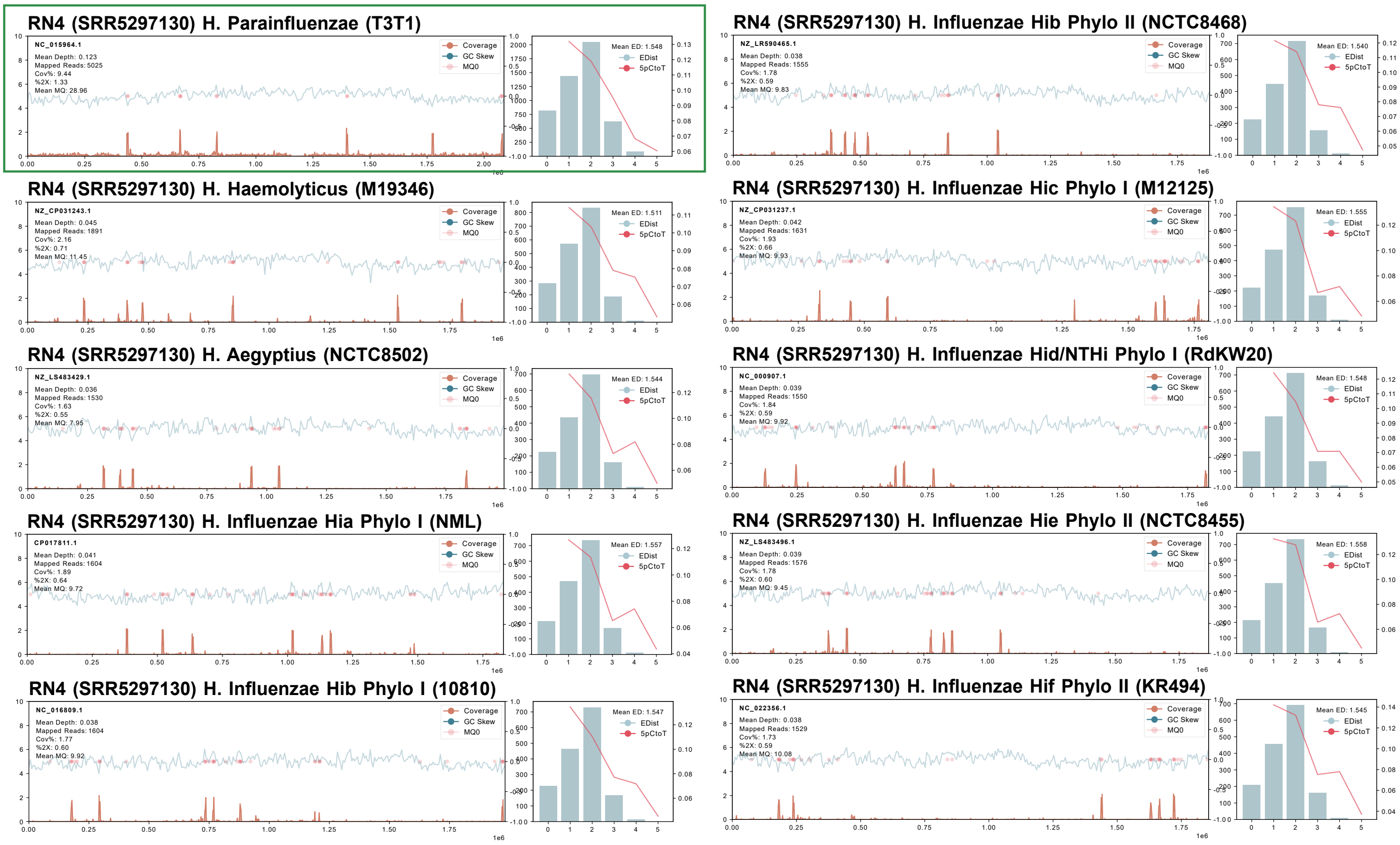

Figure S10: Analysis of calculus sample RN4 from Eerkens et al. 2018.

a) Non-competitive comparative mappings to reference genome for closely related species and serotype a-f. Plots on the left show coverage across the genome, plots on the right depict 5pCtoT deamination signatures (calculated using mapDamage2) and a histogram of read edit distances (-n0.1, MQ>0). The best matching mapping is marked in green.

b) Relative abundance plot for species of the genus Haemophilus for which reads were identified using KrakenUniq (for E-value see methods); generated using plotly.

b)

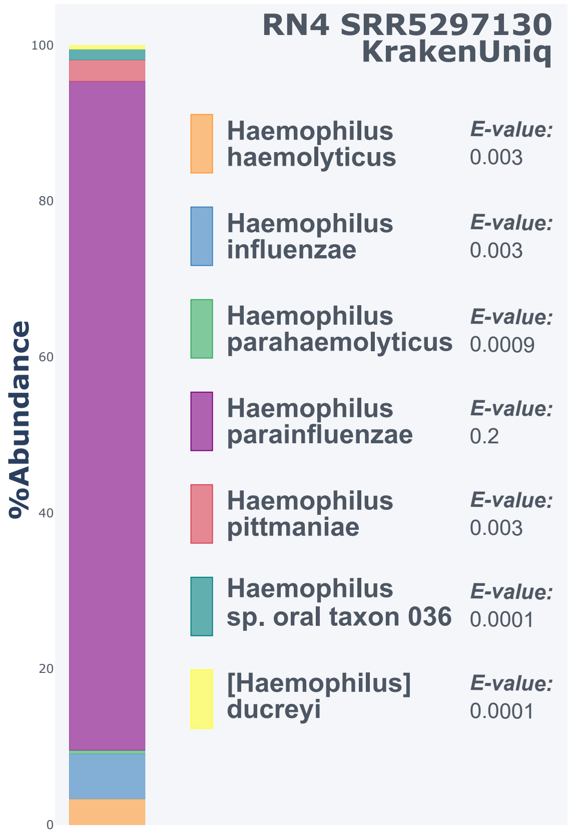

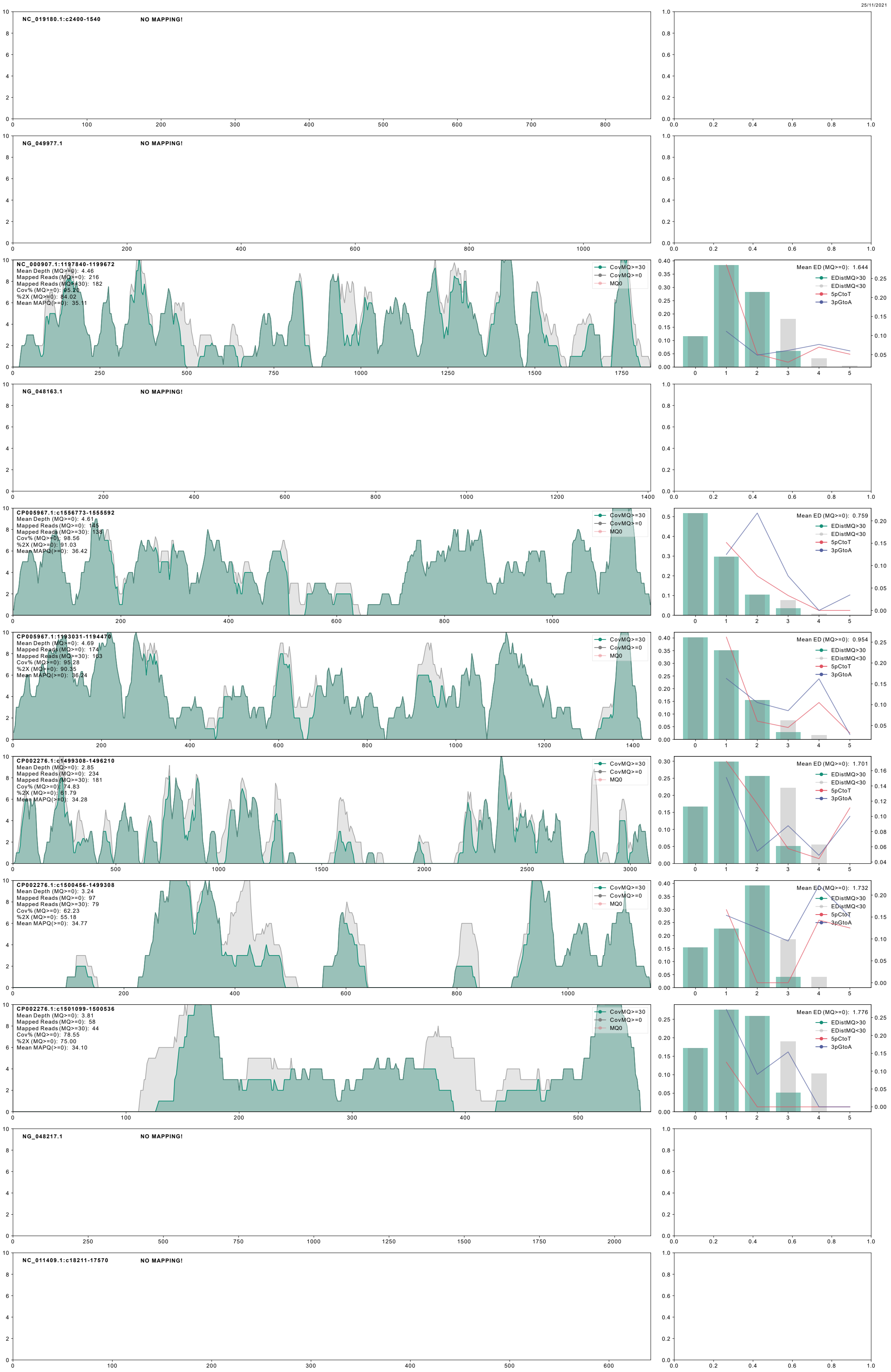

Figure S11: Competitive mapping to the antibiotic resistance reference (see methods). Plots on the left show coverage across the genome, plots on the right depict deamination signatures on the right axis (calculated using mapDamage2) and a histogram of read edit distances.

II. SUPPLEMENTARY TEXT

1. The potential role of *Y. pestis* as enhancer for pre-existing or opportunistic bacterial infections (*Marcel Keller*):

Co-infections of *Y. pestis* with other bacteria are scarcely reported in the scientific literature for multiple reasons. Today, *Y. pestis* outbreaks are more restricted to regions with limited medical capacity for large-scale testing; resources might even be more limited in cases of epidemics [58]. Moreover, the rapid treatment of suspected or confirmed *Y. pestis* infections with streptomycin will likely also suppress pre-existing or acquired co-infections of a broad range of pathogenic bacteria (including *H. influenzae*; cf. [59] and therefore impede their diagnosis. However, during the widespread plague pandemics from Late Antiquity to the Early Modern Era, often affecting immunocompromised populations in the pre-antibiotic era, pre-existing infections or opportunistic superinfections with other bacteria are to be expected.

In modern clinical literature, plague co-infections were described with multi-drug resistant *Stenotrophomonas maltophilia* in a single patient [109] and *Leptospira* in an apparently mixed pneumonia outbreak in the Democratic Republic of Congo [58]. Recently, palaeogenomic analyses also revealed a pre-existing *Treponema pallidum pertenue* infection in a 15th-century plague victim [13]. A previous report of a *Bartonella quintana* co-infection in a 15th-century plague burial from France [110] remains questionable due to the alleged identification of a strain of biovar *Orientalis*, which arguably emerged in the 19th century just prior to the Third Pandemic in Asia [111].

Considering that the first ancient *Haemophilus influenzae serotype b* genome happens to have be retrieved from a plague victim, and the previously published *T. pallidum pertenue* co-infection, a pathogen so far palaeogenomically identified only in two samples from Mexico [112,113] and one from Finland [114], was as well, the question arises whether there is a higher chance of finding other pathogens in plague burials compared to non-plague burials. To investigate this, we discuss three scenarios, which could explain the seemingly increased detectability of other pathogens in plague victims: A) due to higher susceptibility for plague in frail individuals with pre-existing health conditions; B) due to syndemic effects, i.e., synergistic epi-/pandemic spread of two different pathogens in parallel; and C) due to facilitation of bacteremia in the course of a plague co-infection.

Concerning scenario A, it remains a controversial topic in bioarchaeology whether plague was an indiscriminate killer with respect to age, sex and frailty, or not [115]. Whereas some studies could show an increased susceptibility for individuals with pre-existing health conditions [116,117], others could not support that [118]. However, even studies showing increased rates of mortality for frail individuals during plague epidemics when compared to their healthy demographic cohort, do not report more elevated mortality rates than in non-epidemic circumstances [116]. Thus, plague burials might show a higher percentage of frail individuals compared to the living population, but not when compared to attritional cemeteries. Therefore, the likelihood of detecting other pathogens in plague burials does not seem to be elevated by epidemiological factors.

Syndemic effects, as outlined in scenario B, are unlikely to be responsible for the discussed co-infections, since the *T. pallidum pertenue* case likely represents a chronic infection, responsible for periosteal lesions on the skeleton [13], and Hib is expected to be endemic in ancient population. In contrast, plague causes relatively short outbreaks with acute infections, therefore a synchronicity can hardly be established. Syndemic effects are, therefore, more likely to be expected for other acute, epidemic diseases or health conditions such as starvation during famines.

Scenario C, the facilitation of bacteremia of other bacterial pathogens through *Y. pestis*, will be discussed in more detail in the following. Experimental studies concerning co-infections with *Y. pestis* are scarce, but the immunomodulatory effects of *Y. pestis* especially in the first, pre-inflammatory phase are well-known [119]. As shown in a study using trans-complementation assays, the suppression of early innate immune response in pneumonic plague can create a protective environment for microbial proliferation not only of *Y. pestis* pCD1^{-} mutants lacking the type three secretion system (T3SS), but also for other species like *Klebsiella pneumoniae [60]*. Additionally, a *Y. pestis* mutant lacking the plasminogen activator pla could be complemented by its wildtype strain. Remarkably, this trans-complementation for strains missing T3SS could not be reproduced using wildtype *Yersinia pseudotuberculosis* and *K. pneumoniae*, hence we can assume that this anti-inflammatory effect is specific to *Y. pestis* and was not inherited from its evolutionary progenitor, *Y. pseudotuberculosis*. The T3SS is therefore necessary, but not sufficient for this immunosuppression. Although the described trans-complementation was investigated in a pneumonic plague model, the same immunomodulatory effects are expected in other manifestations of plague, which are also characterized by a biphasic progression with a pre- and proinflammatory stage.

As shown by Price et al. [60], the immune suppression of *Y. pestis* can also facilitate proliferation of other bacteria, and might therefore be relevant for co-infections such as the case presented here. This immunosuppressive effect, not attributable to T3SS alone, might also be driven by the plasminogen activator pla. Pla enhances the activation of plasminogen to plasmin directly but also indirectly, e.g. through the inactivation of the PAI-1 (plasminogen activator inhibitor 1, [36], [37]). Plasmin is a serine protease that cleaves fibrin and fibrinogen, excessive plasmin activity, therefore, supports the dissemination of *Y. pestis* through fibrinolysis, which is essential for pneumonic plague [120], but also increases the bacterial load in bubonic plague [121]. Thus, these mechanisms of innate immune system suppression could serve as a model for *Y. pestis'* function as an enhancer of pre-existing or opportunistic infections with other bacteria.

The plasminogen/plasmin homeostasis is, however, a target of many bacterial pathogens. Therefore, the creation of a permissive environment could – depending on the bacterial species involved – be a reciprocal, synergistic process. *H. influenzae* has been shown to acquire plasminogen with a plasminogen receptor, cleaved to plasmin by the host-derived tissue-specific plasminogen activator (tPA). This bacterium-bound plasmin activity occurs in both encapsulated and non-encapsulated strains and also facilitates the dissemination of bacteria through damaging of tissue barriers [122,123].

As an opportunistic pathogen found in the nasopharynx of healthy individuals, *H. influenzae* requires at least a transient bacteremic phase to establish infection foci in other body parts such as joints or the meninges except for non-bacteremic pneumonia. However, once an infection is established, bacteremia can be cleared or drop below diagnostic thresholds, as shown in experimental [124] and clinical studies [125]. *H. influenzae serotype b* is more successful at preventing clearance of bacteremia through the complement system of the host compared to other *H. influenzae* strains, which contributes to its higher virulence [126,127]. Therefore, the individual EDI064 most likely suffered from a pre-existing bacteraemia with *Hib*. Nevertheless, the

co-infection with *Y. pestis* might have facilitated the proliferation of bacteria in the bloodstream and could be one reason for the exceptionally high abundance of *Hib* DNA in the respective sample.

Finally, we considered a combination of scenario A and C specifically for the presented case, where a *Y. pestis* infection creates a permissive environment for proliferation of other pathogens, additionally facilitated by genetic susceptibility for sepsis. Not only *Y. pestis* is known to interfere with the plasminogen system – infections with non-typeable *H. influenzae* have also been shown to induce PAI-1 expression, which inhibits tPA and other plasminogen activators, and therefore plays a crucial role in early host defence [38]. Due to the importance of the Plasminogen activator inhibitor-1 (PAI-1) protein for both *Y. pestis* and *H. influenzae* pathophysiology [36–38]; see discussion), we tested EDI064 for several variants associated with PAI-1 impairment that could have facilitated a *H. influenzae* type b infection at higher than average age and infection with both pathogens in general. The clinical relevance of functional PAI-1 in host defence has been shown for non-typeable *H. influenzae* infection in a mouse model for otitis media [128]. The PAI-1 -675 4G/5G polymorphism, a length polymorphism located in the promoter region of PAI-1, has significant associations with sepsis risk and sepsis mortality [129]. According to ALFA [130] the 4G variant has an allele frequency of around 54% in European populations. A homozygous state (4G/4G) is considered a risk factor for sepsis and numerous other conditions related to fibrinolysis. In EDI064, this position is covered by multiple reads showing both alleles, therefore we assume that this individual was heterozygous. We also tested for two rare variants (43G>A, OMIM: 173360.0003; 2bp insertion 4977TA, 173360.0001) leading to complete PAI-1 deficiency identified in case studies on patients with abnormal bleeding [131,132], but none of them was identified in EDI064. Based on our analysis of loci associated with PAI-1, individual EDI064 did not have an elevated risk of developing sepsis.

In conclusion, while the rapid progression of plague infections and the severity of the osseous changes observed on the skeleton of EDI064 suggest that the *H. influenzae* infection was a pre-existing condition, mechanisms such as the pla-mediated inactivation of PAI-1 through *Y. pestis* could have further contributed to the progression of the *H. influenzae* infection and might have led to additional infection foci. Whether this effect might be observable more generally though, potentially leading to higher detection rates for other pathogens in case of plague co-infections, requires further investigations beyond the scope of this case study.

2. Radiocarbon dating:

A fibula fragment of EDI064 (Sk 447B) was sent to the accelerator mass spectrometry (AMS) facility of the ^{14}CHRONO Centre, Queen's University Belfast, for radiocarbon dating. The retrieved conventional ^{14}C date is 1608±27 BP (UBA-44320) using AMS δ^{13}C correction. Calibration with OxCal v4.4.4 [133] and IntCal20 [134] gave a 2σ interval of 416-541 calAD (see Fig. S9). The range of 416-541 calAD is in line with the archaeological dating and compatible with an association with the Justinianic Plague (541-544 AD), although the 2σ radiocarbon interval (416-541 AD) only overlaps with the onset of the Justinianic Plague as historically reported for Egypt and Palestine [14]. While reservoir effects [135,136] and human bone collagen offset [137] could explain this discrepancy, the δ13C value (-20.7) for EDI064 is consistent with a purely terrestrial diet (δ15N: 10.0). It must also be remembered that although unlikely the date of death could theoretically fall outside the 2σ interval, the 3σ interval for the determination is 383–565 calAD. Precision is impossible but the individual is unlikely to have died after ca. 550, although a death as late as ca. 570 cannot be absolutely excluded.

3. SnpEff analysis:

Our SnpEff analysis yielded six snps with predicted high impact (see Fig S5 & Table S6). A potential start-loss mutation on YfcZ/YiiS, a potential stop-gain mutation on a LysE family transporter (2X T>A, MQ>30) and another potential start-loss mutation on pepT. Additionally, three indels were identified as frameshift variants for a hypothetical protein and genes msbA and a MBL fold metallo-hydrolase (see Fig S5 & Table S6).

4. Eerkens et al. 2018 RN4 re-analysis:

We analysed the dataset available for individual RN4 described in Eerkens et al. [138], for which *H. influenzae* sequences had been described. Data was downloaded from ENA and raw sequencing datasets were trimmed and filtered using cutadapt [64] (-m 30 --nextseq-trim=20 --times 3 -e 0.2 -j 0 --trim-n) in paired end mode (--pair-filter=any) and deduplicated using ParDRe [65]. Trimmed sequences were then merged using FLASH [75] (-M 125 -z). The merged reads were analysed using KrakenUniq as described under "Metagenomics" in the methods. The dataset was also mapped against the same comparative reference sequences as HI-EDI064 (see comparative mappings in methods). Our results (see Fig. S10), show that, based on our metagenomic screening and our comparative mappings, the sequences identified for the genus *Haemophilus* more closely match *Haemophilus parainfluenzae* and are unlikely to be *H. influenzae*. While we might not have identified the most fitting *H. parainfluenzae* reference or potentially species, the mapping clearly shows increased coverage and read counts as well as mapping quality when compared to *H. influenzae*. The presence of *H. parainfluenzae* in dental calculus is unsurprising as it is considered commensal in the oropharynx.

5. Antibiotic-resistance associated loci analysis:

Additionally to our mapping to known *H. influenzae* plasmids (see results & methods), we investigated the presence of loci associated with antibiotic resistance (*ftsl*, *dacA*, *dacB*, *acrA*, *acrB*, *acrR*, *TEM-1*, *ROB-1*, *cat*, *tet(B)* and *tet(M)*) in our sample and found that no genes associated with plasmid-mediated resistance mechanisms were present. However, Penicillin-binding site (PRP3-5) genes, associated with β-lactamase-negative-ampicillin-resistant (BLNAR) strains, and acrABR, associated with the efflux of macrolide antibiotics, showed coverage across most of the gene intervals (~62-98% sequence coverage at a minimum depth of 1) with edit distances ranging from 0.64 to 1.39 when filtering MQ>30 (see Additional file 2: Table S12a and Additional file 1: Figure S11).

We also checked our alignment for the presence of 23 amino acid changes in PRP3 (encoded by *ftsl*), which have been associated with ampicillin resistance (and sometimes also cefotaxime and other cephalosporins), as described in Tristram et al. [139] using reference strain Rd KW20 (see Additional file 2: Table S12b). We detected three amino acid changes consistent with substitutions described for BLNAR strains: V347I (5X, nsSNP 1st Pos G>A), N569S (1X, nsSNP 2nd Pos A>G) and A586S (11X, nsSNP 1st Pos G>T). Two more sites were missing coverage. Substitutions A502T and N526K, which are known to be associated with ampicillin resistance, were not detected and S350N was only detectable at 1X with G>A, which could be due

to deamination. N526K and the R517H, which could be important to phenotypic resistance, were not found. We also checked for the presence of three highly conserved amino acid motives which are essential for PBP3 function [139,140] and found that all were present in our alignment (S327-T-V-K, S379-S-N and K513-T-G). However, it should be noted that we found a shift when comparing the reference. The last amino acid sequence started at aa 512 instead of 513 in our reference, hinting that both this shift and another shift detected for S352S can be associated with the reference sequence (NC_000907.1 Rd KW20). To limit the possibility of off-species mappings, we blasted [141] the reference used for *ftsl* (NC_000907.1:1197840-1199672) using blastn and megablast while excluding hits for *H. influenzae* and filtering for hits with an interval coverage of >80%, but found that all organisms for which hits were present were either closely related species excluded during our species validation steps, synthetic constructs or were not present in our sample based on KrakenUniq output. While it is interesting to find these changes in pre-antibiotic era strains, it should be noted that not the entire interval was covered and coverage was uneven (see Additional file 1: Fig S11), making it hard to evaluate what effect the detected substitution would have had while missing part of the amino acid sequence, particularly when considering the large range, combinations and geographical differences in amino acid changes known to occur [139].

Additionally to PRP3-5 (encoded by *ftsl*, *dacB* and *dacA*), our alignments also show coverage in acrABR intervals. *acrR*, which is important for the efflux of macrolide antibiotics, shows continuous coverage across 78% of the interval but is missing coverage in the first ca. 110 bp and the last 10 bp, which inhibited our inspection of the alignment for early stop mutations caused by insertion induced frameshifts [139]. Finally, we also extracted all reads mapping to the gene intervals using samtools and found that all assignable reads were attributed to *H. influenzae* or a lower taxonomic rank.
