# Supplementary material for: An invasive Haemophilus influenzae serotype b infection in an Anglo-Saxon plague victim
Source: Genome Biol. 2022 Feb 3;23:22. doi: 10.1186/s13059-021-02580-z (PMC8812261; doi:10.1186/s13059-021-02580-z)
Supplement: Supplementary file 3 — Additional file 3. Review history. [file 13059_2021_2580_MOESM3_ESM.docx]

**1^st^ round**

**Reviewer 1**

In this manuscript by Guellil and colleagues, the authors report the detection of Haemophilus influenzae serotype b in an Anglo-Saxon plague victim from circa 540-550 CE using genomic analyses of ancient DNA. The manuscript examines an interesting topic and is well written. The authors provide robust data to support their deduction of a co-infection of H. influenzae and Yersinia pestis in the victim. The statistics in the manuscript, in particular, the Phi test for recombination, appear appropriate. Main comments on the manuscript are as follows:

1. Figure S2 is a key figure which illustrates non-competitive comparative mapping data to support the identification of HI-EDI064 as Haemophilus influenzae. Would therefore recommend including Figure S2 in the main section of the manuscript rather than as supplementary information.

2. The authors examined the genome of HI-EDI064 for the presence of virulence genes. Although the period from which the plague victim EDI064 was buried predates the medical use of antibiotics by more than a millennium, it would be interesting to know if any potential antibiotic resistance genes or mutations are detectable in the genome of HI-EDI064. There are a number of examples of the detection of antibiotic resistance in samples from ancient and remote sources including 5,000+ year-old permafrost (D'Costa et al., Nature 2011; Bhullar et al., PLoS ONE, 2012; Perron et al., PLoS ONE, 2015; Perry et al., Cold Spring Harb Perspect Med, 2016; Pawlowski et al., Nature Communications, 2016).

**Reviewer 2**

An invasive Haemophilus influenzae serotype b infection in an Anglo-Saxon Plague victim (control no. GBIO-D-21-01174)

This manuscript by Guellil et al describes de first identification of an invasive Haemophilus influenzae serotype b strain from the tooth of a six-year-old plague victim over 1,000 years ago. Before the widespread introduction of the serotype b conjugate vaccine, this serotype was indeed the most common cause of invasive disease, but in this case, the Hib strain comes from a co-infection with Yersinia pestis during a Plague Pandemic.

The manuscript is exceptionally well written, methodologically sound and was a pleasure to read. This is one of the best papers I have read as a reviewer, and I had a great time exploring the ancient times. The chronological study is clinically well reasoned and the inclusion of a supplementary text discussing potential co-infection scenarios adds credibility to the findings. Each analysis has been meticulously carried out with appropriate controls.

COMMENT: The ancient H. influenzae strain (HI-EDI064) has been clustered in the phylogenetic division II, close to the Hib NCTC8468, the most phylogenetically different serotype b strain, and not far from the serotype a strains. So close, that "the virulence profile of HI-EDI064 in lipooligosaccharide associated intervals more closely matches the profiles of Hia and Hic-f". Some authors have already defined gradual changes in the H. influenzae capsule genes (Batalha et al, 2010; Terrat et al, 2020). Have you considered the possibility of a capsular switching event?

Minor comments

The NTHi strains (being so heterogeneous) bring a lot of noise to figure 2A, maybe an additional figure could be included with only capsulated strains.

Line 299: Change Streptococcus pneumoniae to S. pneumoniae

Line 320: Change H. influenza to H. influenza

**Reviewer 3**

The authors present an interesting study on the reconstruction of a Haemophilus influenzae serotype b genome in a six-year-old Anglo-Saxon plague victim. The molecular analysis and reconstruction of the Hib genome and the partial Yersinia pestis genome are very well done and convincing. Thereby, the work provides new major insights into the evolutionary history of Hib, a major human pathogen today.

In contrast, the osteological part remains very vague and superficial and does not fully support the conclusions drawn by the authors. However, to support a possible co-infection, it would be necessary to clearly proof the presence of the supposed pathological changes at the inner skull vault and the knee of the child. It is not enough to state that there are osteological signs of a meningeal infection, such as new bone formation at the endocranial surface of the cranium, without providing any further evidence. The same holds true for the supposed septic arthritis of the knees, as neither the ankylosed patellae nor other diagnostic criteria, such as radiographs, CT scans, etc., are shown.

Although the authors clearly state that the osteological could be related to a Hib infection and many other causes, including other bacterial infections and Vitamin deficiencies, they try to direct the interpretation and discussion to a Hib infection.

I think the authors should provide a much more open interpretation and more balanced interpretation of the findings. In particular, they need to present a much more detailed and better documented description of the osteological findings. To achieve it, I would propose the following steps:

1. Provide a more detailed description of the pathological changes with good quality images. In the current manuscript, the endocranial lesions are not shown and the patella anklyosis is not clearly visible in Fig. S1c.

2. Clearly state how the diagnosis of the endocranial lesions and the knees were achieved. What were the diagnostic criteria? Did you use other methods? A differential diagnosis has to be included.

3. Be more cautious with the interpretation. For example, S. aureus is the most common pathogen that causes septic arthritis. This has to be pointed out.

4. There are several publications on the identification and differential diagnosis of endocranial lesions in the paleopathological literature. These studies need to be referenced and taken into consideration.

Based on a more profound osteological analysis, the authors could come to a clearer conclusion, whether they really have identified a co-infection or just the presence of another potential pathogens. In anyway, it remains an interesting finding and offers new clues on the presence of Hib in the past. This is an important finding on its own that doesn't need to be further stressed by a poorly performed osteological analysis.

**Authors’ response**

**Reviewer #1**

1) Figure S2 is a key figure which illustrates non-competitive comparative mapping data to support the identification of HI-EDI064 as Haemophilus influenzae. Would therefore recommend including Figure S2 in the main section of the manuscript rather than as supplementary information.

Response: We have added the figure to the main manuscript.

2) The authors examined the genome of HI-EDI064 for the presence of virulence genes. Although the period from which the plague victim EDI064 was buried predates the medical use of antibiotics by more than a millennium, it would be interesting to know if any potential antibiotic resistance genes or mutations are detectable in the genome of HI-EDI064. There are a number of examples of the detection of antibiotic resistance in samples from ancient and remote sources including 5,000+ year-old permafrost (D'Costa et al., Nature 2011; Bhullar et al., PLoS ONE, 2012; Perron et al., PLoS ONE, 2015; Perry et al., Cold Spring Harb Perspect Med, 2016; Pawlowski et al., Nature Communications, 2016).

Response: We have checked our data for the presence of eleven loci associated with antibiotic resistance and when available checked for specific substitution and indel patterns. We have added an extensive description of our analysis to the SI. A table with coverage and substitution and coverage plots are also available in the SI. A reference to the analysis has been added to the result section.

**Reviewer #2**

1) The ancient H. influenzae strain (HI-EDI064) has been clustered in the phylogenetic division II, close to the Hib NCTC8468, the most phylogenetically different serotype b strain, and not far from the serotype a strains. So close, that "the virulence profile of HIEDI064 in lipooligosaccharide associated intervals more closely matches the profiles of Hia and Hic-f". Some authors have already defined gradual changes in the H. influenzae capsule genes (Batalha et al, 2010; Terrat et al, 2020). Have you considered the possibility of a capsular switching event?

Response: Thank you for pointing this out. We have indeed found that a capsular region III gene hit during serotyping most closely matched Hia genomes as well and have now included the possibility of a capsular switch as an evolutionary scenario for Hib-capsule evolution. We have edited the discussion accordingly.

2) The NTHi strains (being so heterogeneous) bring a lot of noise to figure 2A, maybe an additional figure could be included with only capsulated strains.

Response: We included the NTHI strains in order to have some of their diversity represented in a phylogeny that includes the ancient sample. The second phylogeny which only represents phylogenetic division II does not include NTHi strains for clarity and because the heterogeneity of the NTHi strains reduced the core-snp alignment considerably. The phylogenetic division II phylogeny allows us to use a larger core-snp alignment and thus allows for a clearer placement of HI-EDI064 in the phylogeny. We would therefore ask you to refer to this phylogeny, should the large phylogeny be too noisy.

3) Line 299: Change Streptococcus pneumoniae to S. pneumoniae

Response: This is the first time the organism is named in the manuscript, which is why the genus has been fully spelled out.

4)Line 320: Change H. influenza to H. influenzae

Response: We have amended the spelling.

**Reviewer #3**

1) Provide a more detailed description of the pathological changes with good quality images. In the current manuscript, the endocranial lesions are not shown and the patella anklyosis is not clearly visible in Fig. S1c.

Response: We have added the following to address the concerns over the differential diagnosis ‘There was no evidence for respiratory infection in the form of visceral rib lesions or lytic destruction of the vertebral bodies. There was no porotic hyperostosis, spondylolysis, vitamin D or C deficiency or dental enamel hypoplasia. Cribra orbitalia and sinusitis were unobservable’.

Due to the limitations of diagnosing skeletal lesions, it is not possible to directly attribute the observed lesions to Hib or Yersinia pestis. However, we have added two paragraphs on the diagnosis of the lesions in the knee on page 5 and 6. We have also added references to the literature used in the diagnosis process. We have added a picture of the calcaneus that shows the presence of new bone formation and porosity, as well as an Xray image that shows the posterior aspect of the patellae ankylosed to the femoral epiphysis. We have added a picture of the lesions to the femoral condyles.

2) Clearly state how the diagnosis of the endocranial lesions and the knees were achieved. What were the diagnostic criteria? Did you use other methods? A differential diagnosis has to be included.

Response: A differential diagnosis has been included. Please see pages 5 and 6 of the manuscript. A description of the diagnostic criteria used has also been added. Note the endocranial lesions have been removed because the preservation is just not good enough.

3) Be more cautious with the interpretation. For example, S. aureus is the most common pathogen that causes septic arthritis. This has to be pointed out.

Response: Please see the second and fourth paragraphs of the discussion where we do point out this fact but also stress that clinical literature pre-Hib vaccine indicates that the most frequent cause for septic arthritis at the time was Hib. Following the introduction of the Hib vaccine, S. aureus is indeed the most common cause of septic arthritis. However, as EDI064 was alive pre-vaccine we expect Hib to be the most frequent causative pathogen for septic arthritis in children at the time based on the literature.

4) There are several publications on the identification and differential diagnosis of endocranial lesions in the paleopathological literature. These studies need to be referenced and taken into consideration.

Response: The text that this comment applied to has been removed from the manuscript.

5) Based on a more profound osteological analysis, the authors could come to a clearer conclusion, whether they really have identified a co-infection or just the presence of another potential pathogens. In anyway, it remains an interesting finding and offers new clues on the presence of Hib in the past. This is an important finding on its own that doesn't need to be further stressed by a poorly performed osteological analysis.

Response: Considering that both pathogens were identifiable within teeth, which requires dissemination in the blood, we expect both pathogens to have been active at the same time, meaning that the identification of skeletal lesions would indeed not be necessary to confirm a Hib infection. However, the lesions found on the juvenile skeletons are also consistent with our molecular findings. We, therefore, have two separate independent analytical findings pointing towards a systemic Hib infection. While it is possible another pathogen was at play, H. influenzae is well known to cause infections with similar pathogenic lesions. Had there been a systemic S. aureus infection it seems likely it would have been detectable.

Due to the limitations of diagnosing skeletal lesions, it is not possible to directly attribute the observed lesions to Hib or Y. pestis as; 1) there are no known skeletal lesions associated with Y. pestis and 2) there is no specific diagnostic criteria for Hib infection.
